# Cost hierarchies and the pattern of product cost cross-subsidization: Extending a computational model of costing system design

**DOI:** 10.1371/journal.pone.0290370

**Published:** 2023-09-11

**Authors:** Mark Schmidt, Kai Gustav Mertens, Matthias Meyer

**Affiliations:** Institute of Management Accounting and Simulation, Hamburg University of Technology, Hamburg, Germany; Beijing University of Technology, CHINA

## Abstract

Cost information is critical to ease managers’ decisions in daily business, but its provision is informationally demanding and error prone. Effective design choices for costing systems that can reduce errors are the subject of a growing body of research. The computational model by Anand, Balakrishnan, and Labro (2019) collates previous research in a unifying framework, turning it into a potential standard for future studies. This paper uses this framework and aims to investigate the mechanism behind the well-documented empirical pattern of product cost cross-subsidization in a large-scale simulation experiment. According to this pattern, volume-based costing systems bias the costs of high-volume products upward and of low-volume products downward. Although this pattern has important implications for firms and is discussed extensively in the literature, it has not yet been investigated with computational models. As the first objective of this paper, we replicate the original model by following a pattern-oriented model replication approach. The second objective is to study the mechanism behind the pattern of product cost cross-subsidization. We are unable to reproduce it systematically with the original model. However, the pattern emerges when we extend the model to include a simple cost hierarchy with distinct resource consumption types and volume-based cost drivers. This allows us to specify the likely mechanism behind it. Building on these results, we further extend the model with empirical and theory-based ABC cost hierarchies and assess their effect on product cost cross-subsidization. Our results suggest that production environments underpin more diverse cost hierarchies in practice than previously implemented in the model. Overall, we argue that our extension provides relevant insights into the pattern of product cost cross-subsidization, while our replication and extension strengthen the models’ credibility and usability for future research.

## Introduction

Firms require accurate cost information, especially for decisions on product pricing, product elimination, resource planning, inventory evaluation, and cost management [1–3]. Costing systems report cost information (e.g., costs of products or services) by monitoring resource consumption in production environments. Because products, processes, and organizational structures are manifold and interdependent, costing systems demand data depicting detailed resource consumption. However, obtaining the required data is expensive and often virtually impossible. This results in inaccurate costing information, namely costing errors, as the firm’s costing system does not accurately trace resource consumption. Hence, when designing the costing system, managers face a trade-off to balance the costs of implementing and maintaining detailed and complex costing systems with the negative consequences of costing errors [4].

Against this backdrop, a growing body of research is investigating the effectiveness of alternative design choices in costing systems to reduce costing errors. While certain studies elaborate on individuals’ estimation errors when measuring production activities for costing systems [5,6], other studies employ simulation modeling to capture the interactions between design choices and production environments [7–9] to, in turn, comparatively assess their resulting accuracy. Simulation modeling has the advantage that it can contrast reported cost information with true benchmark costs, which are empirically unobservable [10,11] in a wide variety of production environments. For example, although case studies can investigate design choices in production environments [12], they cannot determine true costs and compare their accuracy with those produced by other design choices.

Anand, Balakrishnan and Labro [13] propose a unifying computational model as a framework (hereafter the ABL framework) to evaluate design choices for costing systems in different production environments. The ABL framework includes design rules to exercise choices on costing systems and embeds model components and results in prior research [e.g., 7–9,14]. This makes it a potential go-to standard for future studies on costing system design to unravel remaining puzzles in practice and literature. For example, there is still no discussion of non-linear cost consumption or the costs of unused capacity [15].

Similarly, large-scale computational experiments have not investigated well-documented empirical patterns like product cost cross-subsidization in volume-based costing systems. This pattern of costing errors is discussed widely in the cost accounting literature and has important practical implications for firms. Accordingly, cost-based pricing tends to distort selling prices due to this pattern: high-volume products would be too expensive, while low-volume products would be too cheap. As a result, the demand for high-volume products might decrease, while low-volume products do not generate enough profit. Overall, this adversely affects a firm’s competitiveness and profitability, emphasizing the practical relevance of this pattern.

This study aims to investigate the mechanism behind the pattern of product cost cross-subsidization by using a large simulation experiment that considers the potential influence of different cost hierarchies. In the spirit of cumulative science, this study pursues a two-step design: first, it replicates the ABL framework; second, it subsequently investigates the pattern of product cost cross-subsidization. Concerning the practice of making the most of computational models, we follow the suggestion of Thiele and Grimm [16] not to build a new computational model from scratch for each research question but (1) to reuse and leverage existing models and (2) to guide the model analysis with empirical patterns [17,18]. Still, computational models and their numerical experiments are prone to programming and implementation errors [19]. Such imperfections are difficult to detect and potentially affect the results of the simulation experiments [20]. A replication is, therefore, imperative to increase the credibility of the computational model’s scientific claims, verify the model’s usability for future studies, and reproduce the findings of this model’s predecessors [e.g., 14,21].

Accordingly, the first objective of this study is to replicate the ABL framework to verify its usability for future studies. To this end, based on the original model’s conceptual description and implementation, we closely replicate the ABL framework by implementing it in a new software environment. We follow prior approaches to computational model replication and adopt best practices [20,22] and guidelines [16,23]. The replication rules out influences on the results caused by implementation specificities or programming errors [20]. We determine replication success by applying the three criteria proposed by Axtell et al. [22]–numerical, distributional, and relational equivalence. These criteria have been applied previously in several replication studies [e.g., 20,24] and are regarded as a quasi-standard. Relational equivalence is achieved when both models qualitatively produce the same results. This criterion has the lowest weight for replication success. Distributional equivalence assesses whether results from both models are statistically indistinguishable [22]. Finally, numerical equivalence specifies that both models compute the same output numerically. Pseudo-random number generators or insufficient sample sizes make it very hard to achieve numerical equivalence for stochastic models [25]. To focus our analysis, we follow the strategy of pattern-oriented modeling [16–18]. We understand patterns as descriptions of specific relations between input and output variables [26] and draw on three well-documented patterns of costing system behavior, namely Cost-pool Relationship, Degree of Resource Sharing, and Dominant Undercosting. We scrutinize whether there are substantial differences between the original and replicated models in regard to the relation described by the pattern.

The second objective of this study is to use the replicated model to dissect the mechanism behind the pattern of product cost cross-subsidization in volume-based costing systems. The product cost cross-subsidization pattern describes that a costing system overcosts high-volume products and undercosts low-volume products [27]. Prior research has already investigated key concepts concerning product cost cross-subsidization. Cooper and Kaplan [28] are the first to note that overhead costs are, in most cases, not proportional to production quantities. They therefore propose Activity Based Costing (ABC) that recognizes and accounts for quantity-independent resource consumption, which they believe is a requirement for the emergence of product cost cross-subsidization pattern.

To support their claim and their newly proposed cost allocation approach, Cooper and Kaplan–in a series of publications [4,27,29–31]–propose the subdivision of a manufacturing firm’s resource consumption into four tiers, which they denote as a “cost hierarchy”: unit-level, batch-level, product-sustaining-level and facility-sustaining-level costs. For instance, unit-level costs vary with activities that occur for single units in the production process (e.g., direct labor). Batch-level costs, by contrast, vary with batch activities (e.g., number of setups). Consequently, allocating costs based on unit-level activities, as in volume-based costing systems, would result in the described product cost cross-subsidization pattern [31]. Therefore, ABC is required to diminish the pattern because it employs activity cost drivers from all tiers of the cost hierarchy. However, although the ABC systems have been substantially developed (e.g., Time-driven ABC [32] and Performance-focused ABC [33]), several surveys still reported a high usage of simple volume-based costing systems [34,35]. Hence, understanding the mechanism behind product cost cross-subsidization in volume-based costing systems is highly relevant for a significant fraction of firms.

Although prior analytical and simulation-based research did investigate costing errors and resulting product cost cross-subsidization, it primarily focused on ABC systems. For instance, Gupta [36] and Labro and Vanhoucke [7] observe the Dominant Undercosting pattern, which results in product cost cross-subsidization for ABC systems. Unlike our experiment, these studies did not consider volume-based costing systems. One prior study that focuses on volume-based costing systems is Hwang et al. [37]. There, the authors develop a numerical example based on an analytical model to study antecedents of over- or undercosting biases in product costs. Although they provide insights into product cost cross-subsidization, their employed numerical example is limited to two products and simple production environments. Other simulation studies [e.g., 9,14] employ the overall accuracy of a costing system as their primary dependent variable and do not focus on product cost cross-subsidization in their analyses. Collectively, previous analytical and simulation studies provide a strong basis for the modeling of various production environments and costing systems. However, since they lack detail or generality, they do not adequately explain the mechanism behind the observed pattern of cross-subsidization of product costs in volume-based costing systems.

We, therefore, aim to investigate the mechanism of product cost cross-subsidization in a large set of production environments representing different cost hierarchies to, in turn, generate a detailed understanding of the pattern in volume-based costing systems. Thus, by following Grimm et al. [17] and implementing additional functionalities and reproducing the pattern of product cost cross-subsidization, we employ a pattern-oriented modeling approach to develop a computational model with greater structural realism.

Collectively, the results of the first objective confirm the successful replicability of the ABL framework. We document relational and distributional equivalence between the original and replicated models. We find that general model behavior holds in our replicated model and, in more detail, we document the reproducibility of the three well-documented patterns drawn from prior research in both models. Concerning the second objective, we show that the unchanged ABL framework does not reproduce the product cost cross-subsidization pattern, indicating that it does not incorporate the mechanism behind this pattern. However, by extending the model with a cost hierarchy of non-unit-level resources and volume-based cost drivers, we can reproduce the pattern, allowing us to specify its likely mechanism based on a large-scale computational experiment. Eventually, we corroborate this mechanism by implementing a full four-tier ABC cost hierarchy and showing that an increased alignment between non-unit-level costs and unit-level cost drivers diminishes the pattern.

## Replication

### Introduction of the numerical framework of ABL

**Fig 1:**
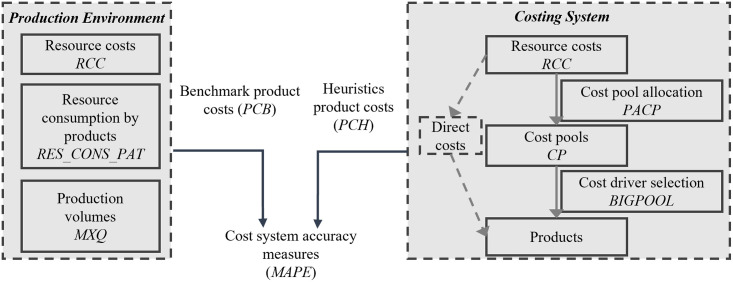
Conceptual illustration of the computational model.

The computational model from the ABL framework consists of two main components: the firm in the form of a production environment and its costing system. The production environment specifies how resources are used to produce products or services. Using a computational model has the advantage that all resource usages are observable without error, that is, a full information setting is provided that, in turn, can be used as a benchmark. This is important in practice, considering that production environments are complex systems involving various interdependencies between machines, labor, administrative tasks, and other supplementary activities. Gathering complete data about such environments is not feasible or simply too costly in practice [38]. Thus, costing systems use limited data when measuring or calculating cost information. This lack of data requires design simplifications and rules of thumb, thus making errors unavoidable. Overall, the model’s objective is to contrast the benchmark of a full information setting of production environments with limited information settings of various costing systems, thereby allowing the calculation of costing system errors. Fig 1 conceptually illustrates the two main components of the computational model.

The first component of the computational model–*the production environment–*is the firm’s full information setting that resembles all resource usages of all products and their production volumes (*MXQ*) in a resource consumption matrix (*RES_CONS_PAT*). The matrix has as many columns as resources (*NUMB_RES*) and as many rows as products *(NUMB_PRO*). Hence, every entry *y* resembles the usage of resource *j* by product *i*. Since every resource has its price, the model computes resource costs (*RCC*) from the *RES_CONS_PAT*. User-defined settings randomly draw all parameters. *MXQ* is drawn from a uniform distribution, with user-specified boundaries to reflect differently heterogeneous production quantities, represented by *Q_VAR*. The resource cost vector (*RCC*) consists of "big" and "small" resources. The input parameter *DISP2* defines "big" resources’ share of the total costs (*TC*), whereas *DISP1* indicates the number of "big" resources, which must not exceed the total number of resources (*NUMB_RES*). Subsequently, a high *DISP2* value in combination with a low *DISP1* value resembles disparate resource costs, with a few "big" resources resulting in a large proportion of the total costs (*TC*). The *RES_CONS_PAT* links production quantities and resource costs. The input parameters *DENS*, *COR1*, and *COR2* generate resource consumption diversity in the *RES_CONS_PAT* matrix. *DENS* defines the number of non-zero entries in the matrix. For example, a value of 0.2 sets approximately 20% of *RES_CONS_PAT* to be non-zero, meaning that products share only a few resources, for example, in a work-shop environment [14]. The correlation parameters set the similarity between resource consumption for two parts of the matrix, aiming to reflect different tiers in the cost hierarchies, such as unit-level and batch-level resources [39]. Therefore, high *COR1* and *COR2* values induce similarity between products through highly correlated resource consumption. Low values increase the disparity, e.g., meaning that products become dissimilar [40]. If all information is available about production volumes (*MXQ*), resource consumption (*RES_CONS_PAT*), and resource costs (*RCC)*, the benchmark costs of a cost object (*PCB*) can be calculated by multiplying a relative resource consumption (*RES_CONS_PATp*) for every resource by every product with the resource costs from *RCC*.


PCB=RES_CONS_PATp*RCC (1)

The second component of the computational model–*the costing system*–only obtains limited information from *RES_CONS_PAT* and then calculates the costs of the final cost objects. The costing system is a two-stage allocation system [7]. First, resource costs are pooled in a selected number of cost pools using a cost-pool-allocation heuristic (*PACP*). Cost pools (*CP*) contain the costs of the pooled resource costs. Second, every cost pool requires an allocation base, called a cost driver, using a cost-driver selection heuristic (*PDR*). The allocation base is the resource consumption of a selected resource. It allocates the cost pool costs to the final objects, assigning the cost information to products, customers, or distribution channels. Balakrishnan, Hansen and Labro [14] find that the choice and functionality of costing system design heuristics (*PACP* and *PDR*) in the two-stage allocation system significantly affect the errors in reported costs.

The original model has four different heuristics for the assignment of resource costs to cost pools, which are described in more detail in the online appendix of Anand et al. [13]:

1. *Size-Miscellaneous* (*SM*): In a setting with m cost pools, the (m-1) largest resources are assigned to one cost pool each. The remaining resources are allocated in the last cost pool (i.e., miscellaneous cost pool, “*miscpool*”).
2. *Size-Correlation-Miscellaneous* (*SMC*): In a setting with m cost pools, the (m-1) largest resources are assigned to one cost pool each. All remaining resources are assigned to these same (m-1) cost pools based on how much they correlate to the seeded resources. Once the total value of the unassigned resources falls below a certain amount of monetary units (*MISCPOOLSIZE*) or the correlation value (*CC*) falls below a certain point, all remaining resources are put in a miscellaneous pool.
3. *Size-Random-Miscellaneous* (*SRM*): The (m-1) largest resources are assigned to (m-1) cost pools. The rest of the resources are then randomly assigned to cost pools until the total monetary value of the unassigned resources falls below a certain amount of monetary units(*MISCPOOLSIZE*). Once this happens, all remaining resources are put in a miscellaneous pool.
4. *Size-Correlation-Miscellaneous-CutOff* (*SCMC*): The largest resource is allocated to a cost pool, then further resources are allocated to this cost pool if their correlation is larger than *CC*. This is repeated for the next cost pools. If there are as many remaining resources as unfilled cost pools, every remaining cost pool is filled with a resource. If more resources are unassigned than empty cost pools and *MISCPOOLSIZE* is reached, every remaining cost pool, except the last, is filled with one resource, and the *miscpool* is filled with the remaining resources.

The *BIGPOOL* method selects a cost driver by finding the largest resource within a cost pool as the cost driver. Because the costing system only obtains a subset of the resource consumption matrix (*RES_CONS_PAT*), it only approximates the full resource consumption. This subset is defined as the activity consumption matrix (*ACT_CONS_PAT*). Each row in *ACT_CONS_PAT* provides the measured resource consumption of the costing system for each cost object and cost pool. Consequently, *ACT_CONS_PAT* has as many columns as the number of cost pools (*CP*). For each cost pool *CP* the sum of the allocated resource costs is known (e.g., from financial accounting [1]). Hence, multiplying the relative resource consumption of every entry with the respective dollar amount allocated in each *CP* provides the occurring costs of each cost object. Summing over the entries for each row provides the reported costs of the cost object (*PCH*) by the costing system.


PCH=ACT_CONS_PATp*CP (2)

As the last step, the Mean Absolute Percentage Error (*MAPE*) between *PCB* and *PCH* of every product *i* is calculated to evaluate the resulting costing errors for different costing system designs in different production environments. S4 Appendix overviews descriptions of all modeled variables and relevant technical terms.


$$MAPE=\sum_{i=1}^{NUMB\_PRO}\frac{{PCH}_{i}-PCB_{i}}{PCB_{i}}$$ (3)

### Replication of the computational model of the ABL framework

The first objective of this study is to replicate the computational model of the ABL framework. The ABL model provides a ready-to-use framework for future research, even though the original paper does not document results. To address relational equivalence, we first conduct a broad numerical experiment–following the 3k-design of experiments–in which we vary all relevant parameters [41]. Although other design of experiment approaches exist in various fields [e.g., 42,43] we orientate along the guideline provided by Lorscheid et al. [41], as it was developed explicitly for simulation-based experiments. Using an OLS regression model containing the relevant variables, we compare their effects on costing errors (*MAPE*) in both models to evaluate relational equivalence. Second, to assess distributional and numerical equivalence between the two models, we focus on three well-documented patterns of costing system design to obtain a relevant angle on the computational model’s results [16,17]. S1 Appendix contains a detailed description of our pattern-oriented replication approach.

Table 1 depicts the conducted numerical experiment with all relevant input, control, and output variables and the factor ranges and levels for the 3k-experiment. We exert a 3k-design parameter setting (’Low’, ’Middle’; ’High’) that upscales the standard 2k-design of ABL (’Low’, ’High’) by evenly separating the parameter range into the three segments. This has the advantage that non-linear effects can be detected [41]. Moreover, for replication purposes, we implement the full range of possible cost pools (i.e., 1 to 50) and all cost pool allocation heuristics (*PACP*) to gain a complete picture of the boundaries. In total, we generate 32,076 design points (i.e., unique parameter combinations) with 729 unique production environment parameter combinations and 44 different costing systems. Combined with the 200 randomly generated production environments (NUMB_FIRMS) described in Anand et al. [13], we obtained 712,800 observations overall. We conduct this balanced design of our experiment for both the original and replicated models [44].

**Table 1:** Design of experiments.

Input variables		Control variables		Output variables
***Production Environment***				
*COR1* *Correlation between volume resources*	U[-0.8,0.8]	*NUMB_PRO* *Number of products*	50	*MAPE* *Mean Absolute Percentage Error*
*COR2* *Correlation between batch resources*	U[-0.8,0.8]	*NUMB_RES* *Number of resources*	50	*BE_AB* *Difference between share of significantly overcosted and share of undercosted products*
*DENS* *Density of RES_CONS_PAT*	U[0.2,0.9]	*NUMB_PRO* *Number of products*		*PCB* *Benchmark costs of a cost object*
*Q_VAR* *Diversity in production quantities*	U[10,20]; U[10,40]; U[10,60]	*CC* *Correlation Cut-off variable*	0.4	*PCH* *Heuristics costs of a cost object*
*DISP1* *Number of "big” resources*	2;5;10	*MISCPOOLSIZE* *Relative share of costs in MISCPOOL*	0.25	*MXQ* *Production quantities per product*
*DISP2* *Share of costs that are assigned to the “big” resources*	U[0.2,0.9]	*TC* *Total Costs*	1.000.000	*Percentage Error (PE)* *PE = (PCH–PCB)/PCB*
***Costing System***		*NUMB_FIRMS* *Number of runs for every input variable combination for the production environment*	200	
*CP* *Number of Cost Pools*	*1*,*5*,*10*,*15*,*20*,*25*, *30*,*35*,*40*,*45*,*50*			
*PACP* *Heuristic to allocate resources into cost pools*	*SM*, *SCM*, *SRM*, *SCMC*			
*PDR* *Heuristic for cost-driver selection*	*BIGPOOL*			

Design points = 11*3*3*3*3*3*3*4 = 32,076 (i.e., unique parameter combinations), 712,800 observations.

### Evaluation of replication success

To evaluate replication success, we compare the replicated model with the original model based on the parameters’ effects on the cost error (*MAPE*). As the different cost pool allocation heuristics (*PACP*) result in quite different costing systems, we split the data set accordingly and conducted an OLS regression for each heuristic. We note that the dependent variables of our regression analyses are not normally distributed in our datasets, which is not unusual for simulation models [45]. Moreover, according to the literature, the normality of the dependent variable is not always necessary to obtain accurate and unbiased estimates in regression analyses, particularly when the sample size is large [46,47]. Nonetheless, to ensure the robustness of our results, we conducted two additional analyses. First, we performed robustness analyses on our regression coefficients with transformations of the dependent variable. Second, we employed a bootstrapping approach for significance testing to address the underlying non-normality. Untabulated results show that our findings remain consistent after both robustness tests. Table 2 provides an overview of the regression analyses for each cost pool allocation heuristic *PACP*.

**Table 2:** Relational equivalence for all parameters.

	SM		SCM		SRM		SCMC	
	*ORIGINAL*	*REPLI CATION*	*ORIGINAL*	*REPLI CATION*	*ORIGINAL*	*REPLI CATION*	*ORIGINAL*	*REPLI CATION*
***Production Environment***								
*DISP1*	0.14**	0.13**	0.11**	0.09**	0.09**	0.09**	0.09**	0.09**
*DISP2*	-0.37**	-0.37**	-0.31**	-0.27**	-0.27**	-0.28**	-0.24**	-0.24**
*DENS*	-0.24**	-0.25**	-0.27**	-0.29**	-0.29**	-0.30**	-0.23**	-0.24**
*COR1*	0.00	0.00	0.00	0.00	0.00	0.00	0.01**	0.00
*COR2*	0.00	0.00	0.00	0.00	0.00	0.00	0.00	0.00
*Q_VAR*	0.00	0.00	-0.03**	0.00	0.00	0.00	-0.04**	-0.04**
***Costing System***								
*CP*	-0.74**	-0.74**	-0.77**	-0.83**	-0.82**	-0.82**	-0.76**	-0.75**
*Adj*. *R*^*2*^	.764**	.763**	.783**	.847**	.848**	.845**	.695**	.691**
*N*	178,200	178,200	178,200	178,200	178,200	178,200	178,200	178,200

Dependent variable: Mean absolute percentage error (*MAPE); CP =* Number of cost pools*; DISP1 =* Number of “big” resources*; DISP2 =* Share of costs that are assigned to “big” resources*; DENS =* Degree of resource sharing*; COR1 =* Correlation between volume resources*; COR2 =* Correlation between batch resources*; Q_VAR =* Disparity in production volumes*;* Presented β coefficients are standardized;

* indicates p < .05.

** indicates p < .01.

First, we note that the adjusted *R*^*2*^ for three of the four heuristics is nearly similar between the original and replicated models. Only for the heuristic *Size-Correlation-Miscellaneous* (*SCM*) does the regression of the replicated model explain more of the total variance, showing a greater difference between the two models. Second, there are two smaller differences in significance levels between the two models. For the heuristic *Size-Correlation-Miscellaneous-CutOff* (*SCMC*), *COR1* has a small significant positive effect on *MAPE*. This effect is, however, only present in the original model. Second, using the heuristic *Size-Correlation-Miscellaneous* (*SCM*), the disparity in production volumes (*Q_VAR*) significantly affects *MAPE* only in the original model (-0.03**) but not in the replicated model. Apart from this, the significance levels are equal for all parameters.

Finally, there are differences at the level of magnitude (e.g., *Size-Correlation-Miscellaneous* (*SCM*)–*DISP1* (original): 0.11**, *DISP1* (replication): 0.09**). There are, however, no changes in the direction of effects between the models. Thus, following Belding [25] that complete equivalence of the original and replicated models is nearly impossible in stochastic simulations, we suggest that the replicated model’s implementation is relationally but not yet numerically equivalent to the original model’s implementation.

### Test of distributional equivalence

To focus our assessment of distributional equivalence, we draw on three well-documented patterns and compare the results of the original and replicated models. Table 3 lists the three patterns and prior references in empirical and theoretical research. The three patterns are: Cost-pool Relationship [e.g., 14,48], Degree of Resource Sharing [e.g., 14,49] and Dominant Undercosting [e.g., 7].

**Table 3:** Three patterns of costing system behavior.

Pattern	Description	Empirical references	Numerical/Analytical References
**Cost-pool Relationship**	*A greater number of cost pools increases the costing accuracy*.	[36,48,50,51]	[7–9,13,14,21]
**Degree of Resource Sharing**	*A lower degree of resource sharing results in a lower costing accuracy*.	[52–55]	[14,21,56]
**Dominant Undercosting**	*Most products are undercosted*.	[36,57,58]	[7,21,59,60]

The perspective on single patterns allows for a more fine-grained analysis of the replication’s success at the level of distributional equivalence. We compare the moments of the distribution of the output variable (i.e., mean, standard deviation, skewness, and kurtosis) for each pattern to evaluate statistical alignment that satisfies distributional equivalence. We purposefully avoid statistical power-tests, such as the Kolmogorov-Smirnov test [22], because statistical tests are over-sensible with large sample sizes [61,62], as in our numerical experiment (712,800 observations). To support our selection, we generate increasing sample sizes randomly drawn from our numerical experiment’s total data set. For each sample size, we compare the Kolmogorov-Smirnov-Test’s p-value and the average deviation in regression coefficients (as in Table 2). S2 Appendix shows this comparison and highlights that the two criteria behave anti-proportional, with the p-value of the Kolmogorov-Smirnov-Test increasing in significance with a larger sample size (indicating differences between the two models), while the regression coefficients become more aligned.

**Fig 2:**
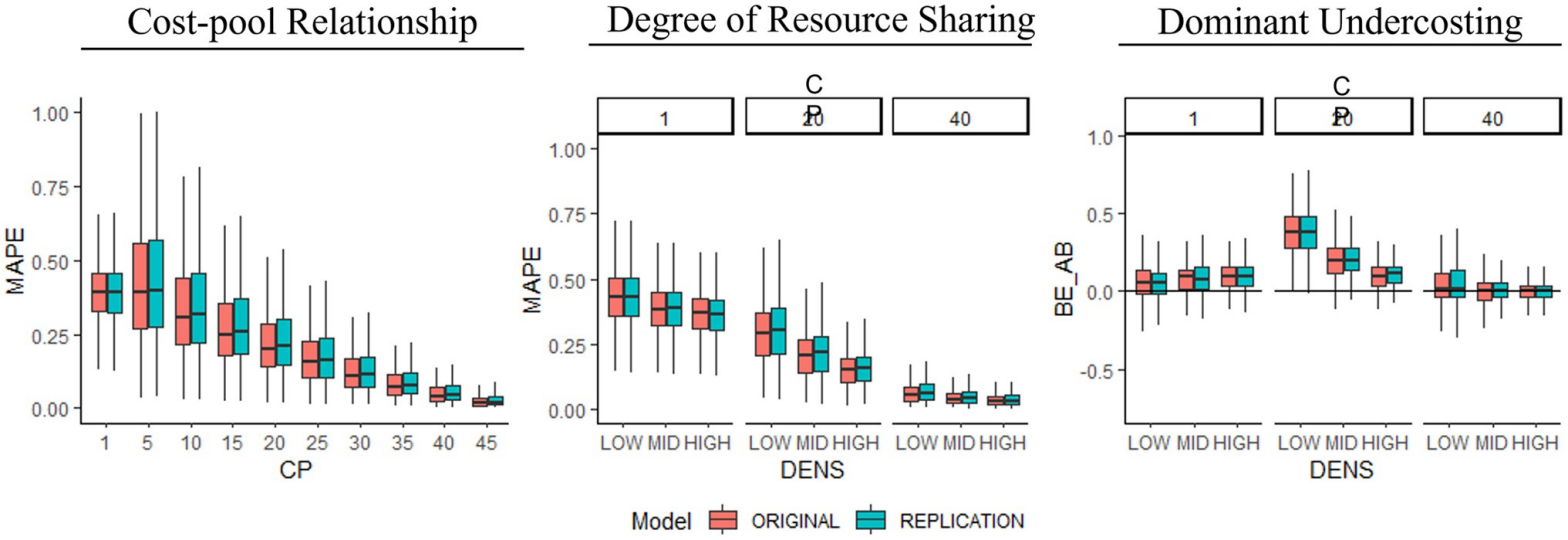
Relational Equivalence Visualizations for the three investigated patterns. MAPE = Mean absolute percentage error. CP = Number of cost pools. DENS = Degree of resource sharing.

The comparison of computed values for the three investigated patterns between the replication model and the original are visualized in Fig 2. The graphs show small but no large differences between the computed values of both models. Note that reproducing the three empirical patterns is not the focus of this experiment but rather the alignment of the two models. However, we find that all three patterns are qualitatively reproduced by both models.

Table 4 lists mean, standard deviation, skewness, and kurtosis for both models’ output variable distributions and for relevant design points in the respective pattern.

**Table 4:** Relational equivalence for the three investigated patterns.

	**Cost Pool Relationship**								
	**REPLICATION**				**ORIGINAL**				
**CP**	**M**	**SD**	**Skew-ness**	**Kurtosis**	**M**	**SD**	**Skew-ness**	**Kurtosis**	**|Δ| M**
1	0.390	0.100	1.54	3.16	0.390	0.099	1.68	3.15	0.000
20	0.235	0.127	1.38	6.40	0.224	0.121	1.37	6.34	0.011
40	0.059	0.049	2.14	9.35	0.055	0.046	2.15	9.32	0.004
	**Degree of Resource Sharing**								
	**REPLICATION**				**ORIGINAL**				
**DENS** ^1^	**M**	**SD**	**Skew-ness**	**Kurtosis**	**M**	**SD**	**Skew-ness**	**Kurtosis**	**|Δ| M**
LOW	0.306	0.130	1.13	5.63	0.301	0.134	1.14	5.59	0.005
MID	0.220	0.100	1.23	6.22	0.217	0.100	1.21	5.89	0.003
HIGH	0.160	0.070	1.19	5.98	0.150	0.070	1.15	5.74	0.010
	**Dominant Undercosting**								
	**REPLICATION**				**ORIGINAL**				
**CP** ^2^	**M**	**SD**	**Skew-ness**	**Kurtosis**	**M**	**SD**	**Skew-ness**	**Kurtosis**	**|Δ| M**
1	0.384	0.090	-0.08	2.92	0.385	0.090	0.00	2.76	0.001
20	0.2	0.11	-0.212	3.41	0.19	0.11	-0.11	3.19	0.010
40	0.014	0.1	1.1	4.9	0.014	0.1	1.1	4.88	0.000

*CP* = Number of cost pools; *DENS* = Degree of resource sharing M = Arithmetic mean of *MAPE*, SD = Standard Deviation of *MAPE*, |Δ| M = absolute difference between the means of REPLICATION and ORIGINAL.

^1^For the values in this table, the number of cost pools *CP* is set to 20.

^2^For the values in this table, the degree of resource sharing *DENS* is set to MID.

For the Cost-pool Relationship pattern, we increase the number of cost pools and observe whether the error in reported costs measured as *MAPE* decreases, as the pattern predicts. In its simplest form, a costing system can be used with one cost pool, which allocates all indirect costs to cost objects as a broad average [63]. Such simple costing systems are considered to be highly inaccurate, causing errors in cost information [63,64]. Increasing the number of cost pools allows for finer assignments of resource costs to cost pools and enables, in turn, a more accurate allocation of their costs. In other words, a costing system with more cost pools captures resources in more detail with more allocation bases [34,53]. Overall, it is common knowledge that adding more cost pools to a costing system increases the accuracy of cost information in most cases [7] because resource consumptions and their costs are more accurately recognized and allocated [7,56]. The absolute differences between the average MAPE of both models are very low for all design points (i.e., different number of cost pools). We understand that both the original model and the replicated model compute statistically comparable results for this pattern.

Second, for the Degree of Resource Sharing pattern, we increase the parameter *DENS*, which specifies the density of the resource consumption matrix (*RES_CONS_PAT*) and therein also determines the diversity in resource consumption along the product portfolio [14]. That is, given a higher degree of resource sharing, cost objects in the portfolio consume resources in similar magnitudes (e.g., marketing efforts are relatively equal for different products). This decreases with a lower degree of resource sharing (e.g., in a job shop environment). In its extreme, no resource sharing would reflect completely unique products, which aggravates accurate cost allocations. Overall, prior research noted that the product diversity created by less resource sharing decreases cost accuracy [14]. Table 4 and Fig 2 show that there are again no larger differences between the original and replication model regarding the Degree of Resource Sharing Pattern. More precisely, the absolute difference between the means for each design point is equal to or less than 1%. Overall, both models reproduce the Degree of Resource Sharing pattern described by prior studies [34,52,54,55].

Third, the Dominant Undercosting pattern included in our replication relates to an effect occurring at the product level when costs are incorrectly allocated to individual products [63]. Prior empirical research and numerical experiments observe that most products are slightly undercosted, while only a few products are largely overcosted [e.g., 7,36]. Please note that a specific form of undercosting of products can also be a deliberate decision, such as when a firm decides not to assign companywide overhead costs to individual products or in a break-even analysis [63]. However, this study focuses on a costing system that aims to allocate all costs to individual products.

To assess the Dominant Undercosting pattern, we follow Labro and Vanhoucke [9] and measure the share of products that are materially undercosted or overcosted and subtract the latter from the former, to construct the measure *BE_AB*. If *BE_AB* is greater than zero, most products are materially undercosted, and the model reproduces the pattern. This measure only considers relevant derivations from true costs, that is, it neglects costing errors below the materiality threshold of errors smaller than 5% [7,65]. The simulation experiment results show that, on average *BE_AB* is greater than zero (see Fig 2), with only a few outliers where *BE_AB* is below zero. More importantly, we again find that both models compute near-similar results for BE_AB at each design point, which are supported by the moments of the distribution of BE_AB in Table 4.

Overall, we conclude that both models behave similarly for all three investigated patterns. We document that our replicated model achieves distributional equivalence to the original model. Hence, in addition to relational equivalence (see Table 2), we consider our replication to be as successful in terms of distributional equivalence for the three patterns.

## Investigation of the pattern of product cost cross-subsidization

### Test of reproducibility in the unchanged computational model

**Fig 3:**
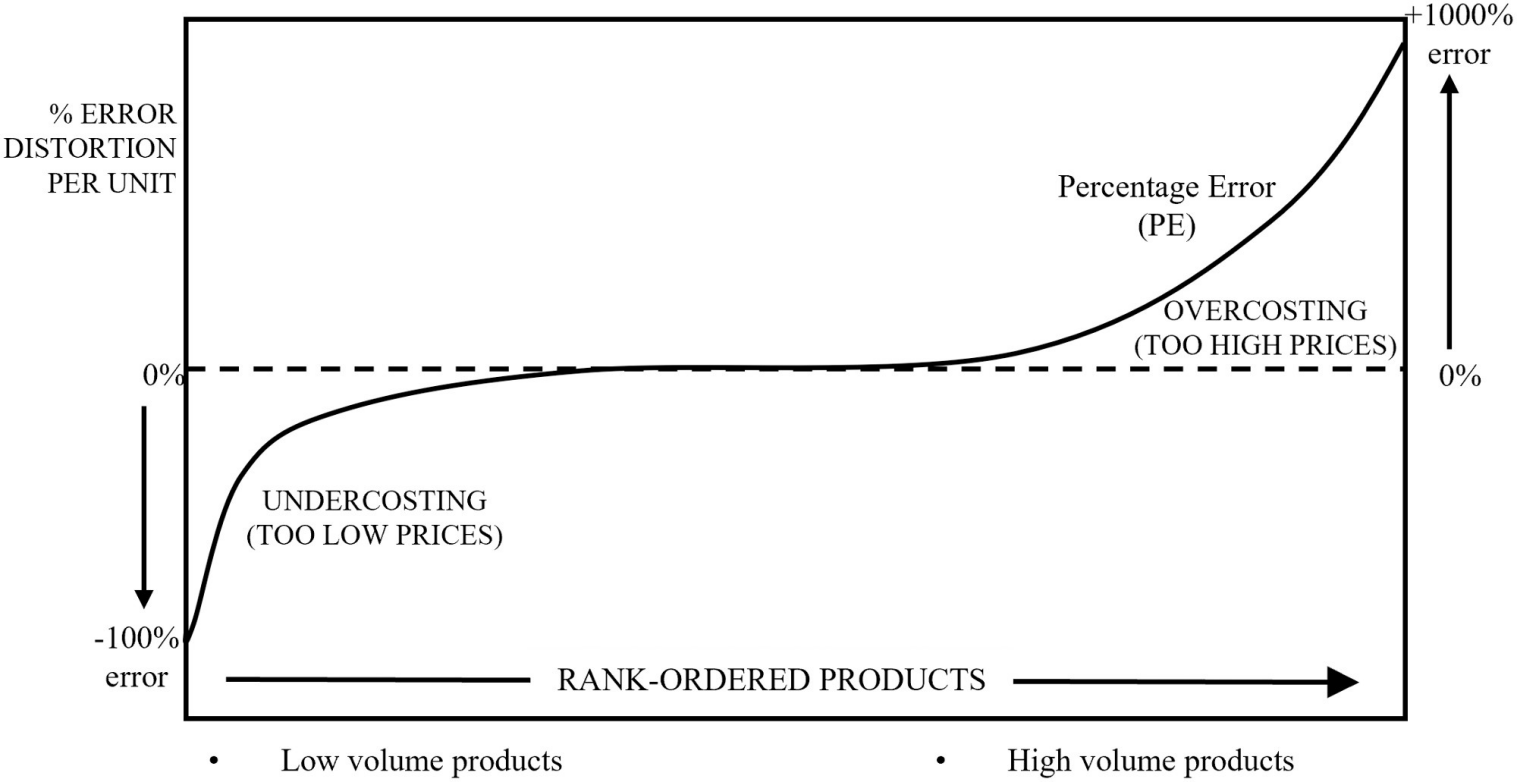
S-Curve of the product cost cross-subsidization pattern.

As the second objective of this study, we investigate whether the replicated model can reproduce the pattern of product cost cross-subsidization (hereafter the pattern) observed empirically for volume-based costing systems [27] and, in addition, examine the required mechanism that ensures the occurrence of this pattern. The pattern shows that, in volume-based costing systems, high-volume products are overcosted while low-volume products are undercosted [63]. Because of the lesser effort required and costs of implementing ABC systems, organizations still use volume-based costing systems [34] (i.e., a single cost driver type and a single cost pool) wherefore the pattern is still likely in these organizations and hence widely shared. The pattern is often depicted as an S-Curve of error, when sorting cost objects along their production volumes (see Fig 3) [66]. Consequently, this pattern negatively affects profits–assuming cost-based pricing–as the demand for the too-expensive products decreases while the demand for the too-cheap products increases [59].

According to Cooper and Kaplan [28], the pattern occurs when the employed cost driver inaccurately reflects true resource consumption by focusing only on unit-level resource consumption (or production volumes). More specifically, such cost drivers do not capture resource consumption that is decoupled from production volumes. For instance, imagine two people having dinner at a restaurant. Person A orders two main dishes, while Person B orders only one. They also decide to share a bottle of wine that costs $30. Person A drinks about one-third of the bottle, while Person B drinks two-thirds. The number of dishes reflects the unit-level resource consumption, while the wine consumption is the non-unit-level resource usage. Note that the wine consumption is decoupled from the number of dishes ordered and even negatively correlates. Nevertheless, a volume-based cost driver might allocate costs for the bottle of wine based on the number of dishes ordered (i.e., unit-level consumption). Consequently, two-thirds of the wine cost ($20) is allocated to Person A and one-third ($10) to Person B because Person A orders two main dishes, while Person B orders only one main dish. Thus, Person A is overcosted (20$>10$), and Person B is undercosted ($10 < 20$). The volume-based cost driver hence ignores non-unit-level resource consumption (i.e., the amount of wine consumed). A solution would be to refine the costing system and to allocate additional costs based on the resource consumption at the other tiers of the cost hierarchy (in manufacturing firms, batch-level, product-sustaining-level, or facility-sustaining-level). In other words, additional cost drivers that measure *non*-unit-level resource consumption are required, such as the number of glasses of wine consumed.

This discussion suggests that two relevant components are required to ensure the emergence of a product cost cross-subsidization pattern. First, at least some proportions of overall costs must be decoupled from volume, meaning that they have a zero or negative correlation to volume. These costs are termed *non-unit-level* costs, while those that are strongly linked to volumes are termed *unit-level* costs. Hence, in the first step, we simplify the four-tier cost hierarchy [31] by converting it into these two segments and argue that non-unit-level costs are necessary for the pattern to occur.

Second, the example also illustrates the requirement that the cost driver of the employed costing system allocates costs based on volumes [27]. A cost driver that additionally considers non-unit-level resource consumption will diminish the pattern [27,56]. More generally, a refined costing system employing cost drivers that allocate costs based on resource consumption, which reflects all present levels in the firm’s cost hierarchy, should prevent the occurrence of the pattern [67]. This conception led to the development of ABC [62], where the cost drivers in the costing system ideally measure resource consumption on all tiers of the cost hierarchy. Case studies on firms observe that ABC shifts reported costs of high-volume products downward and costs of low-volume products upward [4,57], indicating the reduction of the product cost cross-subsidization pattern. Still, the pattern and its mechanism remain uninvestigated in settings that exceed the limit of numerical examples or single cases.

**Fig 4:**
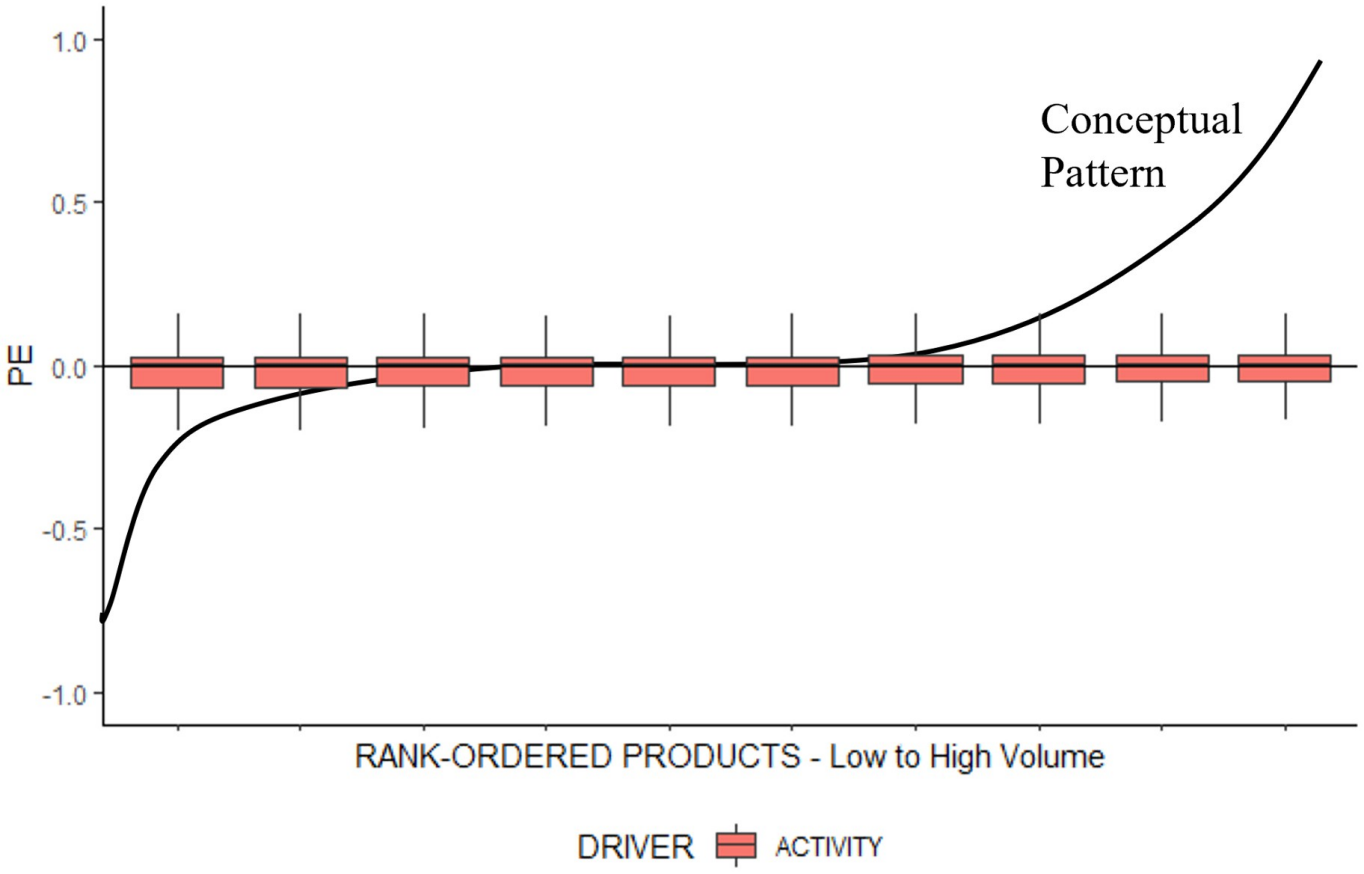
Product cost cross-subsidization pattern in the replicated model.

For the replicated model, we expect that the pattern of product cost cross-subsidization will not emerge, because (1) the model currently only computes resource consumption that is highly correlated with production volume (see S3 Appendix) and further employs an activity-based cost driver that would considers non-unit-level resource consumption [14]. To test this assumption, we recreate the S-Curve in Fig 3‘s conceptual illustration with the data generated by the replicated model. For each run, we group all products into deciles based on their production volumes and calculate the percentage error *PE* for each product. According to the pattern, the lowest-volume (highest-volume) deciles should have a negative (positive) percentage error. Fig 4 shows that, for the replicated model, there is no systematic distortion along the rank-ordered products, suggesting that the pattern cannot be reproduced.

### Extension of the computational model to reproduce the pattern

To reproduce the pattern, we implement the two components as suggested: the volume-based cost driver and non-unit-level costs. First, for the volume-based cost driver, we use production volumes (i.e., *MXQ*) as the allocation base for a product’s overall resource consumption. We recognize that other cost drivers, such as direct labor and machine hours, exist for volume-based costing systems, which are also related to production volumes or production-linked activities [59]. However, in our modeling approach, we have opted for simplicity and used production volumes as the sole cost driver type. An exploratory analysis conducted in Mertens [64] indicates high similarity between different volume-based drivers, further justifying our approach.

Second, to model non-unit-level costs, we divide the resource consumption matrix (*RES_CONS_PAT*) into unit-level and non-unit-level resources (the columns in the matrix). As in the original model, the unit-level resource consumptions are multiplied by the production volumes. This step results in highly correlated resource consumptions with production volumes, defined as unit-level consumptions. S3 Appendix illustrates that when all resources are multiplied by production volumes, the median correlation between resource consumption (*RES_CONS_PATp*) and production volumes (*MXQ*) is above 0.75. This correlation decreases with an increased share of non-unit-level costs, showing that the non-unit-level costs in the model correlate less with *MXQ*. Based on the findings of Ittner, Larcker and Randall [39], who observe that roughly 40% of activities are *not* on the unit-level, we randomly set 20% to 60% of all resources and costs to the non-unit level. For our experiment, we divide this simple cost hierarchy into four specifications, depending on the share of non-unit-level resources–(1) all resources are on the unit-level and their consumption correlates with production volumes (as in the original model), (2) 20%-33% of all resources are non-unit-level (LOW), (3) 34%-46% of all resources are non-unit-level (MID), and (4) 47%-60% of all resources are non-unit-level (HIGH). We control for all possible costing system designs and production environment parameters (as in the replication experiment presented in Table 1).

**Fig 5:**
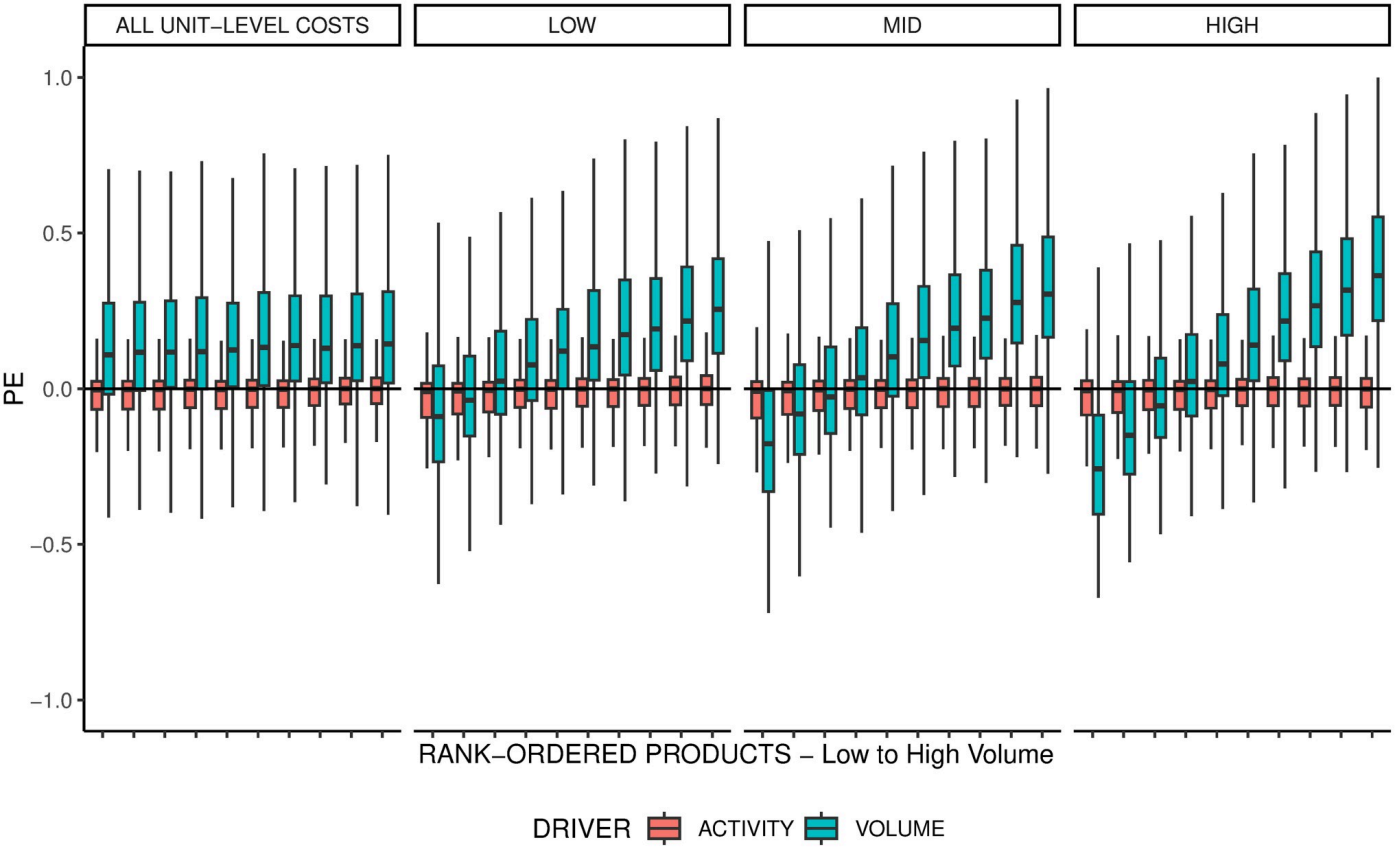
Product cost cross-subsidization pattern for volume-based costing and activity-based costing. LOW = Share of costs that are consumed on the non-unit-level = 20% - 33%; MID = Share of costs that are consumed on the non-unit-level = 34% - 46%; HIGH = Share of costs that are consumed on the non-unit-level = 46% -60%. For the volume-based driver the mean values for VB_PATTERN in the three types of cost hierarchies are: ALL UNIT-LEVEL COSTS = 0.03; LOW = 0.31; MID = 0.45; HIGH = 0.58.

Fig 5 illustrates the results for the different settings of the numerical experiment with the four specifications of the simple cost hierarchy and the two types of cost drivers (VOLUME and ACTIVITY). Both a volume-based cost driver and non-unit-level costs are required to reproduce the pattern. More specifically, in these treatments, low-volume products are likely to be undercosted and high-volume products are likely to be overcosted, as described by the pattern. Furthermore, a greater share of non-unit-level resources strengthens the pattern, as shown in Fig 5. This corroborates that the expected mechanism is driving the pattern and that the simple cost hierarchy containing two types of resource consumption (i.e., unit-level and non-unit-level) is sufficient to reproduce the pattern. On a different note, our results highlight that ABC systems are unaffected by the presence of non-unit-level resource consumption regarding (1) the product cost cross-subsidization pattern and (2) overall accuracy of reported product costs. Fig 5 illustrates that the percentage error PE for all products in the portfolio is similar. Moreover, PE is close to zero in all settings (i.e., the size of the boxplots is small), indicating a low overall error in reported product costs as well. This result underscores the superiority claim of ABC advocates [4]. To quantify the pattern, we compute the variable *VB_PATTERN*. *VB_PATTERN* is the difference between the mean percentage error *PE* for the products in the two highest and lowest production volume deciles. In other words, we calculate the difference between the two extreme right-hand boxes and the two extreme left-hand boxes in the boxplots of Fig 5. Hence, the greater the value for *VB_PATTERN*, the greater the difference between the two groups, and the greater the strength of the pattern (i.e., the steeper the S-Curve). We observe that in volume-based costing systems *VB_PATTERN* increases as the share of non-unit-level resources increases.

Case studies that observe the pattern report a usage of volume-based cost drivers [4,57,58], which is in line with our suggested mechanism. Based on our literature review and the work of Anderson and Sedatole [67], empirical research is still inconclusive about the existence of cost hierarchies. Since non-unit-level resource consumption is required for the emergence of the pattern–according to the identified mechanism–we argue that this hints at the existence of at least one tier in the cost hierarchy (e.g., batch-level) in empirical production environments.

As an added analysis, we conduct a regression analysis to measure the effects of other variables on the variable *VB_PATTERN*. Table 5 depicts the direct and interaction effects of the input variables on *VB_PATTERN* in an effect matrix [41]. The binary variable *VolumeDriver* indicates whether the employed cost driver is based on production volumes (*VolumeDriver* = 1) or activities (*VolumeDriver* = 0, *BIGPOOL*, see Table 1). The variable *non_unit_size* provides the share of resources (i.e., columns in *RES_CONS_PAT*) that is not multiplied with production quantities and hence are on the non-unit level (20% - 60%).

**Table 5:** Effect matrix for *VB_PATTERN*.

	Factors								
Factors	*CP*	*DISP1*	*DISP2*	*DENS*	*COR1*	*COR2*	*Q_VAR*	*Volume* *Driver*	*non_unit* *_size*
*CP*	-0.02**	0.00	0.00	0.00	0.00	0.00	-0.01**	0.02**	0.00
*DISP1*		0.00	-0.01**	-0.01**	0.00	0.00	0.00	-0.01**	-0.01**
*DISP2*			0.01**	-0.01**	0.00	0.00	0.00	0.04**	0.03**
*DENS*				-0.05*	-0.01**	-0.01**	0.00	-0.04**	0.01**
*COR1*					0.01**	0.00	0.00	0.01**	0.00
*COR2*						0.01**	0.00	0.01**	-0.01**
*Q_VAR*							0.12**	0.11**	0.12**
*Volume Driver*								0.38**	0.36**
*non_unit_size*									0.37**
*R*^*2*^ = .457** N = 316,800									

Dependent variable: *VB_PATTERN*; *CP =* Number of cost pools*; DISP1 =* Number of “big” resources; *DISP2 =* Share of costs that are assigned to “big” resources; *DENS =* Degree of resource sharing; *COR1 =* Correlation between volume resources; *COR2 =* Correlation between batch resources; *Q_VAR =* Disparity in production volumes; *VolumeDriver* = Indicator variable for the usage of volume-based cost driver (1) or activity-based cost driver (0); *non_unit_size* = share of resources that are non-unit-level; Presented β coefficients are standardized;

* indicates p < .05.

** indicates p < .01.

Intuitively, the presence of a volume-based cost driver (*VolumeDriver*) and an increase in non-unit-level costs (*non_unit_size*) have the greatest effect on the emergence and strength of the pattern (0.38** and 0.37**, respectively). Additionally, the interaction effect between these two variables is substantial (0.36**), indicating the importance of the interplay of the two model components to reproduce the pattern. Moreover, Table 5 reports that the disparity in production volumes within the product portfolio, measured by *Q_VAR*, has a strong positive effect on *VB_PATTERN*. This also aligns with our explanation of the pattern’s mechanism. The greater disparity in production volumes results in a volume-based cost driver that more strongly overestimates (underestimates) the non-unit-level resource consumption of high-volume (low-volume) products. Returning to our restaurant example, this means that Person A would eat four dishes while Person B would eat only one. Consequently, products with extremely high or low production volumes are significantly more affected by cross-subsidization when a volume-based cost driver is used.

**Fig 6:**
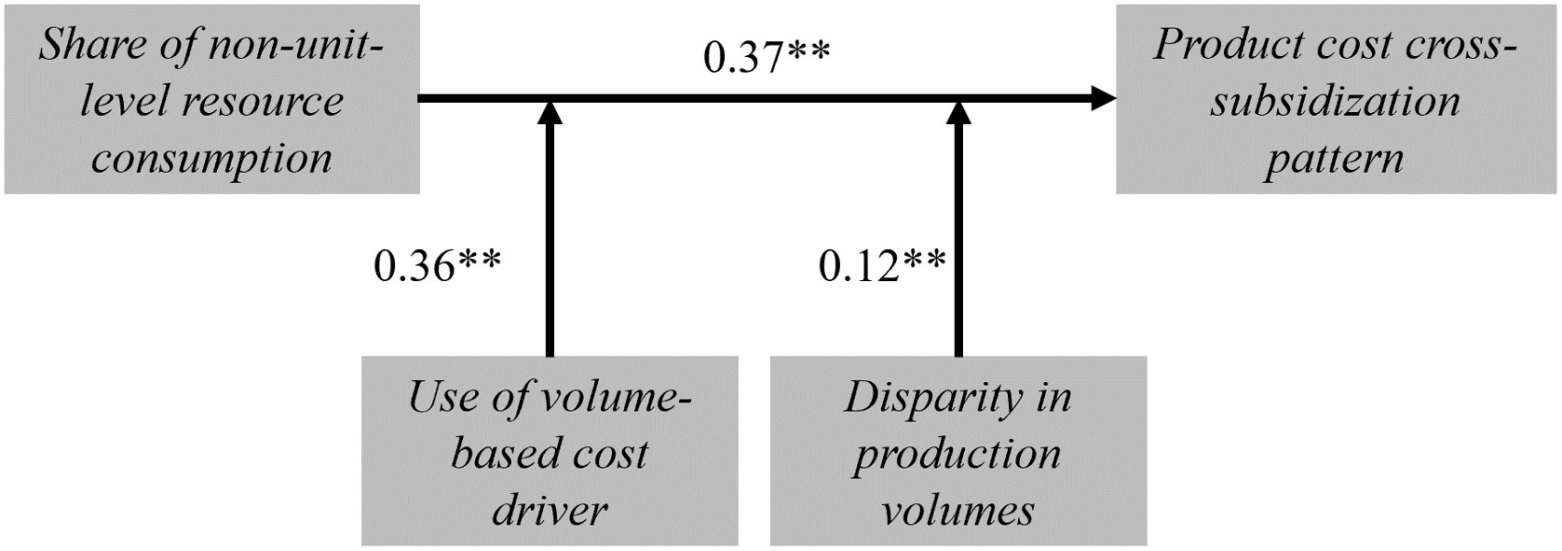
Mechanism of the product cost cross-subsidization pattern. The product cost cross-subsidization pattern is measured using the variable VB_PATTERN; Disparity in production volumes is measured by Q_VAR; Use of volume-based cost driver is the indicator variable for usage of volume-based cost driver (1) or activity-based cost driver (0); Share of non-unit-level resource consumption is measured using the variable non_unit_size; Presented β coefficients are standardized; * indicates p < .05. ** indicates p < .01; N = 315,600.

Summarizing our results, we can now depict the mechanism behind the pattern of product cost cross-subsidization. Fig 6 shows the main effect of the share of non-unit-level resource consumption on the pattern (*VB_PATTERN*) and the moderating effects of using a volume-based cost driver and disparity in production volumes.

A split of the sample into costing systems with and without volume-based drivers (see S1 Fig) shows that a strong relationship between the share of non-unit-level resource consumption and the pattern of product cost cross-subsidization (0.60** vs 0.03**) is only apparent in costing systems with volume-based cost drivers. Similarly, the interaction effect of the disparity of production volumes is much stronger in volume-based costing systems (0.18** vs 0.03**). This analysis further supports the identified mechanism.

### Extension of the computational model by an ABC cost hierarchy

Although the implementation of non-unit-level costs that do not vary with production quantities suffices as a simple cost hierarchy to reproduce and explain the mechanism behind the pattern of product cost cross-subsidization, it does not represent a full ABC cost hierarchy as theoretically proposed [31]. In a full ABC cost hierarchy, a distinction is made between unit-level, batch-level, product-sustaining-level, and facility-sustaining-level costs. In our current modeling approach, we model resource consumption that varies with production volumes (unit-level) and non-unit-level consumption that varies randomly. In the following experiment, we investigate whether a further separation of non-unit-level costs into batch-level, product-sustaining-level, and facility-sustaining-level costs affects the emergence and strength of the product cost cross-subsidization pattern.

Based on the literature, we first argue that our approach of modeling non-unit-level costs most likely represents facility-sustaining activities, such as plant management [31], because there is no significant link to unit-level production activities. Second, batch-level costs vary with batch-related activities, such as the number of batch setups or setup time [67], and are, according to economic-order-quantity theory (EOQ), negatively associated with production quantities [68]. That is, batch sizes increase with greater production quantities, which reduces batch-level activities per production unit [69]. Batch-level resource consumption, therefore, negatively correlates with unit-level resource consumption and production volume. Finally, product-sustaining resource consumption is linked with activities that depend upon product variety, complexity, and resulting production process activities, such as process design [31,67]. Product-sustaining activities ought to correlate slightly with production quantities [39], as product-sustaining activities are linked with variable production depending on the type of employed manufacturing technology (e.g., Advanced Manufacturing Technology vs Workshop Production) [67].

In addition to the theoretical construction of the ABC cost hierarchy provided by the literature, we draw on the empirical stream of accounting research that provides empirical evidence on cost hierarchies and the linkages between different tiers of the cost hierarchy. The empirical observations differ from the theoretical predictions. Therefore, we distinguish between *empirical* and *theoretical* in our modeling approach, with the former indicating the results that are more likely to be observed in practical settings. Table 6 provides the described theoretical predictions of links between the different tiers of the ABC cost hierarchy (-;0;+) in the upper diagonal cells and depicts the empirically reported Pearson correlations between tiers in the lower diagonal cells. As described, the empirical observations are inconclusive and display a wide range of observed correlations. For instance, the correlation of resource consumption between unit-level and batch-level activities ranges from insignificant (e.g., 0.07 [39]) to significantly high (e.g., 0.82** [70]).

**Table 6:** Empirical evidence on correlations between different tiers of the ABC cost hierarchy.

Tier/Tier	Unit-level	Batch-level	Product-sustaining-level	Facility-sustaining-level
**Unit-level**	1	**-**	**0; +**	**0**
**Batch-level**	**0.07** [39] 0.02–0.82**[70] **0.05–0.10** [71]	1	**0; -**	**0**
**Product-sustaining-level**	**0.19** [39] 0.43**– 0.78**[70]	-0.41**[39] 0.30**- 0.93**[70] 0.12**[72]	1	**0**
**Facility-sustaining-level**	0.57* [73] **0.08–0.20** [71]	0.28**[72] **-0.17** [73] -0.30**– 0.20 [71]	0.69**[72] 0.44* [73]	1

* indicates p < .05.

** indicates p < .01, as found by the original studies.

We select the following modeling approach to insert the two types of the ABC cost hierarchy (*theoretical* and *empirical*) into the resource consumption matrix (*RES_CONS_PAT*). First, we continue modeling unit-level costs as in the original model and the model with the *simple cost hierarchy* in the previous experiment by multiplying randomly drawn resource consumption *λ* with production volumes *q* to generate highly correlated unit-level costs *y*. Second, to model batch-level resource consumption for the *theoretical* ABC cost hierarchy, we divide the randomly drawn normal distributed resource consumption *λ* for each batch-level resource and product by the production quantities *q* of the respective product. Hence, greater production quantities result in larger batch sizes and decreased batch-level costs *y* per produced unit (i.e., negative correlation). Third, to reflect the *empirical* ABC cost hierarchy, we model a weak positive correlation between unit-level and batch-level resource consumption by multiplying the random resource consumption *λ* with the respective production quantities *q* and a random number *f* drawn from a normal distribution with mean = 1 and standard deviation = 0.25.

Next, the product-sustaining-level resource consumption is modeled by multiplying the product of random resource consumption *λ* and production quantities *q* with the factor *r* drawn from a normal distribution with mean = 1 and standard deviation = 0.25. The random value *r* can be seen as the type of manufacturing technology that either couples or decouples product-sustaining activities from unit-level activities (e.g., Advanced Manufacturing Technology vs Workshop Production) [67] and thus decreases or increases its linkage and correlation. Moreover, in the theoretical setting, a positive correlation between unit-level and product-sustaining-level resource consumption results in a negative correlation between batch-level and product-sustaining-level resource consumption, as posited by Ittner et al. [39]. Overall, for product-sustaining-level resource consumption, we do not distinguish between a *theoretical* and *empirical* modeling approach, as observations and theoretical predictions align.

**Fig 7:**
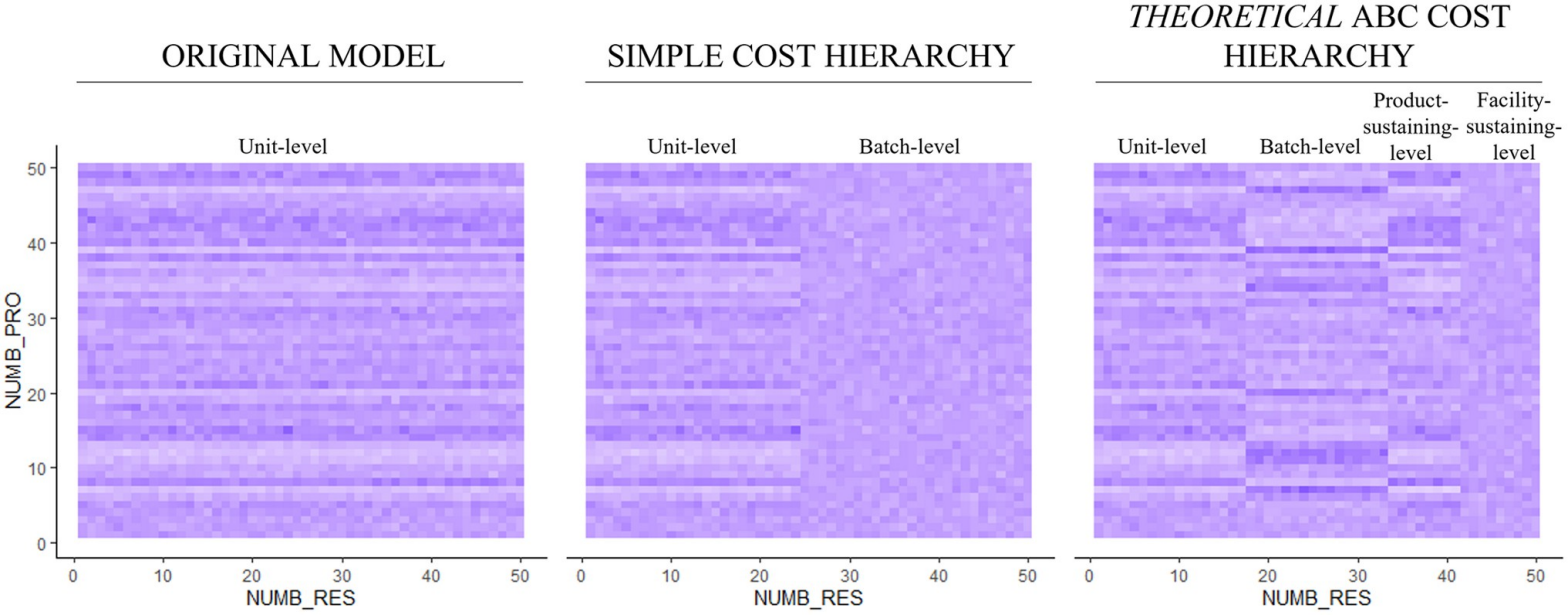
Exemplary visualization of resource consumption patterns with different types of cost hierarchies. Each row in the matrix reflects the relative resource consumption of the respective product. Each column in the matrix reflects how one resource is consumed by all products (rows). NUMB_PRO = Number of products in the firm’s portfolio (50). NUMB_RES = Number of resources consumed (50).

Finally, in the *simple cost hierarchy* (prior section), we generated the facility-level costs *y* for each product and respective resource solely from a random resource consumption *λ*. This resulted in resource consumption without a significant correlation between facility-level resource consumption and other tiers of the ABC cost hierarchy. This also reflects the theoretical intuition concerning facility-level costs [31]. However, empirical accounting research also observes strong positive (and some negative) correlations between facility-level costs and all other tiers of the ABC cost hierarchy. Hence, to relax the strict decoupling from other tiers of the ABC hierarchy, we multiply the randomly drawn resource consumption *λ* for the facility-level resources with one of the respective weighting factors (i.e., *q*, *r*, or *f*) of the other tiers. We randomly select which factor *λ* is multiplied to provide a basis for all possible scenarios. Fig 7 exemplarily illustrates how the resource consumption matrix (*RES_CONS_PAT*) contains different tiers of resource consumption when the *simple cost hierarchy* or the *theoretical* ABC cost hierarchy is introduced, compared to the original model. Resource consumption is less homogeneous among all resources because it does not solely correlate with production quantities.

To define the share of resources and costs that fall within the respective tier of the cost hierarchy, we rely on our modeling for the simple cost hierarchy and set 20–60% as non-unit-level costs (i.e., batch-level, product-sustaining-level, and facility-sustaining-level costs). Moreover, in their case study, Cooper and Kaplan [31] find that about 20% of the associated costs are batch-level costs, 18% are product-sustaining costs, and 7% are facility-sustaining costs. We orientate along these observations and model the following shares of tiers in the ABC cost hierarchy to add up to 100%: unit-level = 40%– 70%, batch-level = 10%– 32%, product-sustaining-level = 10%– 24%, and facility-sustaining-level = 5%– 15%. Table 7 reports the Pearson correlations for resource consumption between the different tiers of the four modeled cost hierarchies. Overall, we pursue the approach to model a variety of cost hierarchies to cover different industry settings, strategic orientations, and production technologies to increase generality of our results. For instance, Advanced Manufacturing Technologies can shift resource consumption from batch-level and product-sustaining-level toward unit-level or facility-sustaining-level [67]. Supply chain design (i.e., distance to supplier or sales markets) may determine logistics efforts, thus increasing batch-level costs [2]. A firm’s strategic orientation affects research and development efforts [74] or product design [75] and may shift costs toward facility-sustaining- or product-sustaining-level costs. Anderson and Dekker [2] and Banker et al. [76] review prior findings on how such factors influence costs and resource consumption.

**Table 7:** Pearson correlations (and standard deviations in brackets) for the resource consumptions between the different tiers of the implemented cost hierarchies.

		ORIGINAL MODEL			
Tier	Modeling	Unit-level	Batch-level	Product-sustaining-level	Facility-sustaining-level
Unit	*y = λq*	1			
Batch	*y = λq*	.46^1^ [0.22]	1		
Product-sustaining	*-*	-	-	1	
Facility-sustaining	*-*	-	-	-	1
		SIMPLE COST HIERARCHY			
Unit	*y = λq*	1			
Batch	-	-	1		
Product-sustaining	-	-	-	1	
Facility-sustaining	*y = λ*	.15^2^ [0.22]	-	-	1
		THEORETICAL ABC COST HIERARCHY			
Unit	*y = λq*	1		-	-
Batch	*y = λ/q*	-.53 [0.23]	1		
Product-sustaining	*y = λqr*	.43 [0.23]	-.38 [0.21]	1	-
Facility-sustaining	*y = λ*	.00 [0.15]	.00 [0.14]	.00 [0.13]	1
		EMPIRICAL ABC COST HIERARCHY			
Unit	*y = λq*	1		-	
Batch	*y = λqf*	.44 [0.21]	1		
Product-sustaining	*y = λqr*	.42 [0.19]	.32 [0.17]	1	-
Facility-sustaining	*y = λ[q][r][f]*	.00 [0.14]	.09 [0.20]	.09 [0.23]	1

All correlations are significant with p < 0.01. N = 240,000 observations. Standard deviations are reported in square brackets.

*y =* cost consumption; *λ =* random resource consumption drawn from a normal distribution with mean = 1 and standard deviation = 0.25; *r* = normal distribution with mean = 1 and standard deviation = 0.25; *f* = normal distribution with mean = 1 and standard deviation = 0.25.

^1^Note that in the original model, Anand et al. (2019) generate one section of the resource consumption matrix to reflect batch-level resources by employing the input variables *COR1* and *COR2*. However, all resource consumptions *λ* are multiplied by production quantities.

^2^In the simple cost hierarchy modeled in the previous section, the non-unit-level costs are modeled without linkage to production quantities, wherefore we classify them here as facility-level costs.

**Fig 8:**
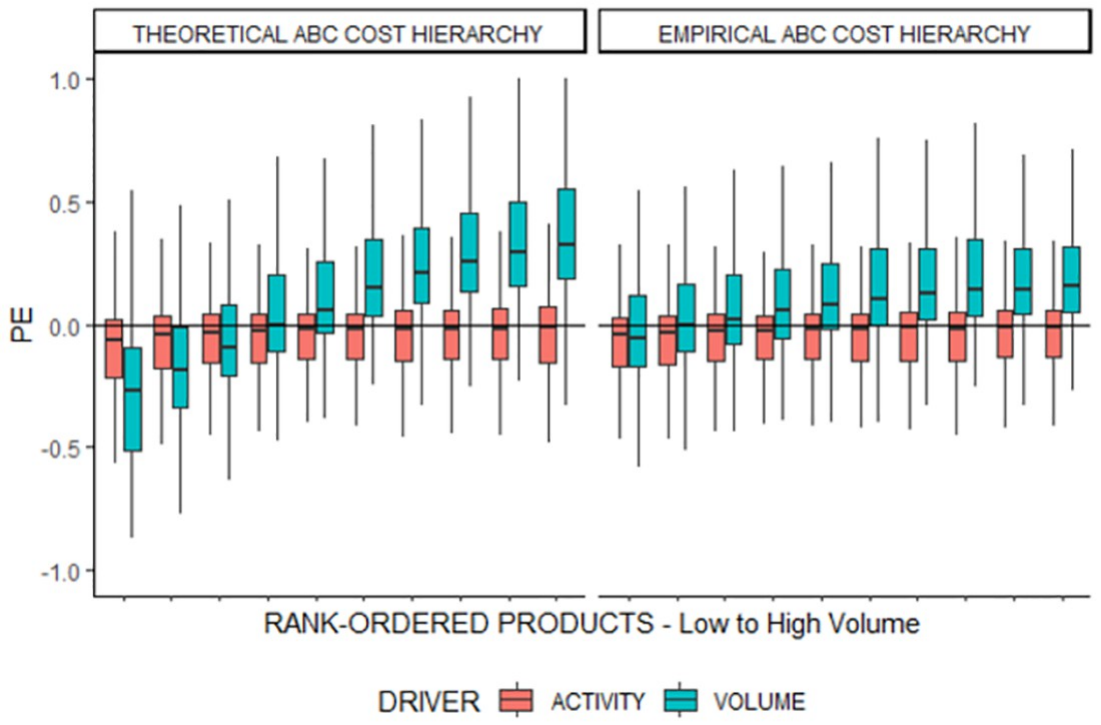
Product cost cross-subsidization pattern in the theoretical and empirical ABC cost hierarchy. PE = Percentage Error between reported product costs (PCH) and true benchmark product costs (PCB). For the volume-based driver, the mean values for VB_PATTERN in the two ABC cost hierarchies are: Theoretical = 0.40; empirical = 0.14.

According to the reasoning behind the ABC cost hierarchy, negative correlations should be a stronger driver of the pattern than no correlations because the former reflects anti-proportional resource consumptions that contradict the costs reported by employed cost drivers [56], as in our restaurant example. Hence, we assume that the *theoretical* ABC cost hierarchy produces the strongest product cost cross-subsidization pattern. The results of our third simulation experiment support this assumption. Fig 8 illustrates the product cost cross-subsidization pattern for firms with *theoretical* and *empirical* ABC cost hierarchies. The pattern does not emerge as pronounced in empirical ABC cost hierarchies, although there is a small overcosting bias toward high-volume products. However, as expected, the cross-subsidization is strongest in the *theoretical* ABC cost hierarchy.

This strengthens our argument and theoretical predictions [37] that the correlations between the different tiers’ resource consumption are critical for the pattern to emerge. Consequently, as the empirical ABC cost hierarchy contains relatively high positive correlations, the cross-subsidization is weak, whereas in the theoretical ABC cost hierarchy, resource consumption can be negatively correlated, and the cross-subsidization is strongest. This may hint at a divergence between theoretically expected and empirically observed cost hierarchies and resulting product cost cross-subsidization. Despite this, we argue that empirically it is difficult to attain correlations between different resource consumptions. A reason is that the true resource consumption pattern is not empirically measurable [10]. Researchers must rely on employed cost drivers containing aggregation, specification, and measurement errors [64]. Additionally, Cooper and Kaplan [31] posit that when non-unit-level resource consumption is divided by unit-level cost drivers, the impression of high correlation can arise. Finally, we again employ the variable *VB_PATTERN* to quantify the drivers of product cost cross-subsidization. Table 8 reports the regression results for the four different cost hierarchies.

**Table 8:** Regression analysis for VB_PATTERN in the four cost hierarchy models.

Predictor	ORIGINAL MODEL	SIMPLE COST HIERARCHY	THEORETICAL ABC COST HIERARCHY	EMPIRICAL ABC COST HIERARCHY
**Production Environment**				
DISP1	-0.03**	-0.03**	-0.05**	-0.07**
DISP2	-0.02**	0.01**	0.12**	0.12**
COR1	-0.01**	-0.01**	0.00	0.00
COR2	0.02**	-0.01**	0.00	0.00
DENS	-0.03**	-0.01**	-0.11**	-0.10**
Q_VAR	0.05**	0.23**	0.24**	0.16**
bl_size	0.00	-	0.08**	0.02**
pl_size	-	-	0.02	0.01**
fl_size	-	0.12**	0.04**	0.10**
**Costing System**				
CP	-0.01**	-0.01**	0.00	-0.00
*Volume Driver*	0.03**	0.53**	0.56**	0.32**
*R* ^*2*^	.006**	.350**	.412**	.170**
*Mean*	0.02	0.23	0.40	0.14

Dependent Variable = *VB_PATTERN*; *CP =* Number of cost pools; *DISP1 =* Number of “big” resources; *DISP2 =* Share of costs that are assigned to “big” resources; *DENS =* Degree of resource sharing; *COR1 =* Correlation between volume resources; *COR2 =* Correlation between batch resources; *Q_VAR =* Disparity in production volumes; *VolumeDriver* = Indicator variable for the usage of a volume-based cost driver (1) or activity-based cost driver (0); *bl_size* = share of resources that are batch-level; *pl_size* = share of resources that are product-sustaining-level; *fl_size* = share of resources that are facility-sustaining-level; Presented β coefficients are standardized;

* indicates p < .05.

** indicates p < .01.

The *R*^*2*^ is highest for the *theoretical* ABC cost hierarchy because resource consumption follows systematic rules in that setting; therefore, the resource consumption matrix (*RES_CONS_PAT*) is the most structured. In turn, the regression models of the original model and the *empirical* ABC cost hierarchy can only explain smaller fractions of the variation of *VB_PATTERN* because resource consumption is more randomly generated (see Fig 7 as an example). Interestingly, due to structuring the resource consumption matrix into more than two tiers (i.e., for the *theoretical* and *empirical* ABC cost hierarchies), the parameters *DISP2* and *DENS*, in particular, become more relevant for cross-subsidization. *DISP2* primarily defines the heterogeneity of resource costs. Hence, in a more structured matrix where some resource consumptions are not proportional to production quantities, heterogeneous resource costs can be a lever to increase the cross-subsidization when production quantities are employed as a cost driver. In other words, allocating costs based on production volume is especially detrimental (with high cross-subsidization) when a few non-unit-level resources contain a large share of costs. This principle also applies to *DENS*, which determines the degree of resource sharing. The greater the degree of resource sharing, the more homogeneous the resource consumption along different hierarchy tiers, resulting in a less pronounced pattern. Collectively, these results suggest that distinguishing between different levels and types of the cost hierarchy further contributes to a better understanding of the pattern.

## Conclusion and discussion

In this paper, we investigated the mechanism behind the pattern of product cost cross-subsidization in a large-scale simulation experiment based on a replication of the computational model developed by Anand, Balakrishnan, and Labro (1). To ensure the accuracy of our replication, we followed the best practices of computational replications, including building on the conceptual model underpinning the original model and detecting potential implementation and programming errors. We also employed a pattern-oriented modeling strategy to guide our more detailed analyses within the original and replicated models and their behavior [17]. We selected three well-documented empirical patterns to test the distributional and numerical equivalence of the models’ outcomes. We compared the statistical moments of the distributions of interest to assess their likeness. We propose this approach as it is more straightforward and robust than the traditional statistical tests used to test distributional equivalence (e.g., t-tests or Kolmogorov-Smirnov-test), which are deemed problematic considering the large sample sizes typical for simulation experiments. While numerical equivalence was not achieved (as expected for models with several stochastic components), we found distributional equivalence for all patterns. In sum, our results verified relational and distributional equivalence between the original and replicated models, thus confirming replication success.

Next, we used the replicated model to investigate the mechanism behind the pattern of product cost cross-subsidization in volume-based costing systems. The original model was not designed to reproduce this pattern, and the initially implemented production environment only created unit-level costs that correlated highly with production quantities. To reproduce the observed pattern, we extended the model by adding two new components: a volume-based cost driver and non-unit-level costs. Our findings revealed that both components must be incorporated to successfully reproduce the pattern, suggesting that a more diverse production environment, including non-unit-level costs, is required for product cost cross-subsidization in volume-based costing systems. By using a large-scale simulation experiment, we were able to analyze the mechanism underlying the pattern in detail, identify the key variables involved, and quantify their relationships. Our results showed that dispersed production quantities and a high share of non-unit-level costs increase the pattern’s strength in volume-based costing systems. Therefore, in such settings, the managers of firms should exercise prudence while making decisions based on costs.

To better differentiate the impact of non-unit-level costs, we extended our model in a second way by implementing two complete four-tier ABC cost hierarchies. This allowed us to explore the impact of different cost hierarchies on the pattern of product cost cross-subsidization, thereby gaining a more detailed understanding of how the pattern emerges in different production environments. Our approach to investigate the full effect of the four-tier ABC cost hierarchy on the pattern of product cost cross-subsidization is based on empirical observations and theoretical predictions. We derive a pattern-orientated modeling approach to achieve this. The results show that the pattern diminishes when batch-level, product-sustaining-level, or facility-sustaining-level resource consumption does not have a zero or negative correlation with unit-level cost drivers, corroborating the identified mechanism. While the pattern of cross-subsidization has been observed in several case studies [12,77], our identified mechanism suggests that both empirical and theoretical ABC cost hierarchies can produce the pattern. However, our findings indicate that the theoretical hierarchies with negative correlations are especially critical in generating this pattern and may exist in the production environments of certain firms for which the pattern has been reported [4,12].

Our study makes two significant contributions to the literature. First, we contribute to simulation-based and analytical accounting research on costing system design and accuracy by successfully replicating the ABL framework and extending it with a volume-based cost driver and different cost hierarchies. By doing so, we complement investigations that were limited to a few examples [37] or that focused on the product cost cross-subsidization pattern of ABC systems [7]. Our study also contributes to the discussion of volume-based costing, which is still widely used in practice [34]. Additionally, our modeling approach can be useful when investigating decisions and practices that require more detailed cost hierarchies, such as customer or product profitability analysis. Such insights can be particularly relevant and add a new dimension to existing studies. This underscores the importance of measuring the cost hierarchy in practice when investigating cost-based decision-making. Overall, our study contributes to the discussion on costing system design and provides useful insights into cost accuracy and errors in reported product costs.

Second, our study also contributes to the discussion of cost hierarchies in accounting research [67]. Specifically, we synthesize empirical observations with theoretical predictions about resource consumption correlation to develop a modeling approach for different cost hierarchies. By linking simulation-based research on costing system design with empirical research on ABC cost hierarchies [39], we leverage the advantages of simulation modeling to examine the conditions under which cost hierarchies are less likely to result in product cost cross-subsidization in volume-based costing systems. This information can be valuable when estimating the potential occurrence of product cost cross-subsidization in practice. In addition, our research offers a fresh perspective on cost driver research [78] by investigating the emergence of the pattern of product cost cross-subsidization in both theoretical and empirical ABC cost hierarchies. We detail this understanding by reviewing prior empirical findings of cost hierarchy characteristics and allow for a more explicit linkage to specific types of cost hierarchies and their effect on the pattern of product cost cross-subsidization.

On a different note, our investigation does not come without limitations. First and foremost, we scrutinize the mechanism behind the pattern of product cost cross-subsidization in a simplified simulation model. Our approach may neglect confounding factors present in empirical studies, as well as how managerial action influences how resources are consumed over time. Hence, it is important to consider the impact of managerial action and the resulting timely perspective on cost hierarchies, as other theories on cost behavior suggest [76]. We, therefore, encourage future research to investigate the effect of such characteristics on the pattern of product cost cross-subsidization. Furthermore, because empirical proof of the existence of a cost hierarchy is limited [70], contemporary cost accounting argues that the traditional fixed and variable cost structure may be more accurate [67]. Since we did not incorporate this in our investigation, further research is needed to better understand the relationship between activities and overhead costs. Nevertheless, we believe that our findings will provide a solid stepping-stone for such considerations and guide future empirical and simulation-based research on this topic.

## Supporting information

S1 Fig Product cost cross-subsidization mechanism for costing systems with and without a volume-based cost driver. The product cost cross-subsidization pattern is measured using the variable VB_PATTERN; Disparity in production volumes is measured by Q_VAR; Share of non-unit-level resource consumption is measured using the variable non_unit_size; Presented β coefficients are standardized; * indicates p < .05. ** indicates p < .01; N = 158,400 for each model.
(TIF)

S1 Appendix Detailed description of applied replication methodology. (DOCX)

S2 Appendix Additional analysis: Kolmorogov-Smirnov-Test with increasing sample sizes. (DOCX)

S3 Appendix Additional analysis: Correlation between resource consumption and production volumes. (DOCX)

S4 Appendix Overview of variables and technical terms in the simulation experiments. (DOCX)

S5 Appendix Supporting information for data and code availability. (DOCX)
