# Supplementary figures and images for: Cost hierarchies and the pattern of product cost cross-subsidization: Extending a computational model of costing system design
Source: PLoS One. 2023 Sep 11;18(9):e0290370. doi: 10.1371/journal.pone.0290370 (PMC10495028; doi:10.1371/journal.pone.0290370)

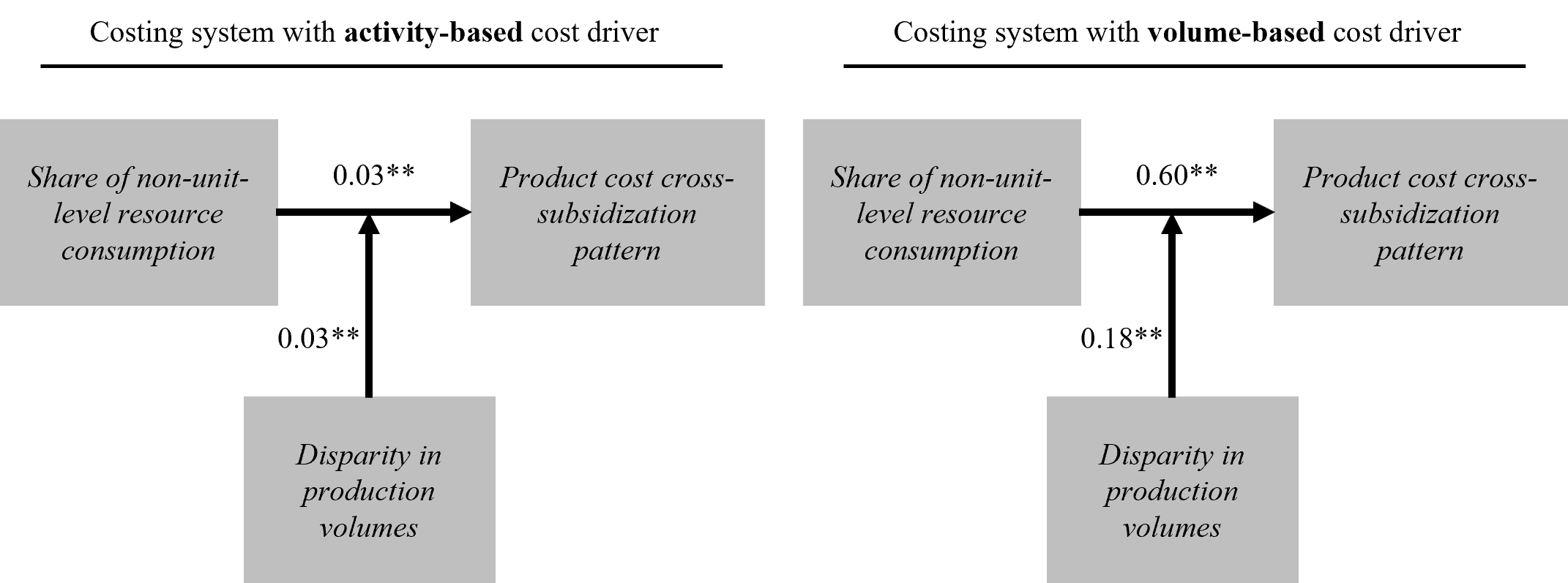

Supplement: S1 Fig — The product cost cross-subsidization pattern is measured using the variable VB_PATTERN; Disparity in production volumes is measured by Q_VAR; Share of non-unit-level resource consumption is measured using the variable non_unit_size; Presented β coefficients are standardized; * indicates p < .05. ** indicates p < .01; N = 158,400 for each model. (TIF) [file pone.0290370.s001.tif]
