# Supplementary material for: Cost hierarchies and the pattern of product cost cross-subsidization: Extending a computational model of costing system design
Source: PLoS One. 2023 Sep 11;18(9):e0290370. doi: 10.1371/journal.pone.0290370 (PMC10495028; doi:10.1371/journal.pone.0290370)
Supplement: S1 Appendix — (DOCX) [file pone.0290370.s002.docx]

# S1 Appendix

# Methodology

This paper investigates the mechanism behind the pattern of product cost cross-subsidization. In the first part of the study, we replicate the ABL framework and assess the replication success. In the second part, we investigate the pattern of product cost cross-subsidization. Using the replicated ABL framework, we test whether the model reproduces the pattern and substantiates the likely mechanism behind it. Fig 1 depicts the two-step approach of this study.





**Fig 1. Design of the study.**

## Replication of the original model

In the first step of our approach (see Fig 1), we replicate the computational model of the ABL framework by following best practices [1, 2]. We construe the replication of a computational model as its reimplementation in a different modeling software [3]. This reimplementation is ideally based on the conceptual model [4] of the original computational model (i.e., model description) to rule out the implementation and programming errors of the original model that can influence the results [2]. For example, implementation errors are misinterpretations of the conceptual model, including its theories or mathematical formalisms. Textual explanations in the model description may not be sufficiently clear to everyone, thus creating space for different interpretations and implementations of the model. Programming errors are not innocuous but can sometimes drive results more than the actual model [5]. In our study, the replication is based on the conceptual description and code of the ABL framework. These are provided in the online appendix of Anand, Balakrishnan and Labro [6].

Two issues, however, affect the replication's feasibility if strictly limited to the conceptual model [1]. First, ambiguities in writing or formalities in the conceptual model might be too great, preventing a replication from the conceptual model only [7]. Second, as a more extreme scenario, a conceptual model could contradict the actual implementation of the original model [8]. For example, mathematical formalisms do not converge between the conceptual model and the code. Thus, it is important to note and specify ambiguities and contradictions during the replication process.

The replicated model must be compared with the original model along predefined criteria to evaluate replication success. Most models answer particular research questions [9] and produce results that facilitate answer-based conclusions [10]. Computational models apply simulation experiments that can be used to compare the original and replicated models. The replication succeeds if both models produce identical results in different dimensions [11].

Some computational models, however, are published as frameworks without explicit experiments and results, as is the case with the ABL framework. Its purpose is to encompass previous models' key elements and support future research endeavors. As compensation for the lack of specific results, we follow the suggestion of Thiele and Grimm [10] to use empirically observed patterns to guide the replication. Since the ABL framework includes previous models, it is reasonable to argue that it could reproduce earlier studies' patterns. Given the underlying model, we understand patterns as descriptions of specific relations between input and output variables [12, 13]. Thus, we focus on the patterns from earlier studies that ought to be reproducible with the ABL framework. We draw on three well-documented patterns of costing system behavior, namely Cost-pool Relationship, Degree of Resource Sharing, and Dominant Undercosting. The three patterns each address a different aspect of the model (costing system design, production environment, errors in single product costs), hence, providing a comprehensive view of model behavior.

To evaluate replication success, Axtell et al. [14] propose applying three assessment criteria – relational equivalence, distributional equivalence, and numerical equivalence – to the results of the original and the replicated model. These criteria have been applied previously in several replication studies [e.g., 2, 9] and are regarded as quasi-standard.

Relational equivalence is achieved when both models qualitatively show the same relations between input and output variables [14]. This relational equivalence can be understood as a qualitative reproduction of a result. Still, it might be the case that the direction of a pattern is reproduced but not its magnitude [15]. Unsurprisingly, the relational equivalence of computational models has the lowest weight for replication success.

Distributional equivalence is achieved when both models produce statistically indistinguishable results. The different statistical methods that are used to test distributional equivalence (e.g., t-tests or Kolmogorov-Smirnov-tests) [11] usually assess whether two sets of measures (e.g., relevant results from both models) can be drawn from the same distribution [14]. Despite their simple application and reasonability to check distributional equivalence, Secchi and Seri [16] note that traditional statistical tests are likely to report irrelevant differences as significant when sample sizes are large. Thus, tests of statistical significance may be flawed with many observations typical for simulation research [17].

Finally, numerical equivalence is achieved when both models numerically produce the same results. That is, both models compute precisely the same numerical values in the same experiment. In settings with a few simulation runs, random numbers may cause numerical differences between the dependent variables of two experiments. In settings with sufficient runs, different random-number generators may cause deviations. As they only draw pseudo-random numbers, they create systematic differences in the dependent variables of the two experiments. Hence, in stochastic computational models, it is challenging to achieve numerical equivalence [18].

Collectively, assessing replication success using these criteria provides an overview of whether a reimplementation in different software (ideally based on the conceptual model) can reproduce the original model's results and underlying mechanisms. The replication increases the internal validity of the computational model while simultaneously ensuring the absence of programming and implementation specificities (such as unintended errors) that affect results.

## Investigation of the reproducibility of the empirically observed pattern

In the second step of our approach (see Fig 1), we address the replicated model's ability to reproduce the empirically observed pattern of product cost cross-subsidization in volume-based costing systems. It should be noted that the model was not explicitly designed for this purpose. Accordingly, we must introduce a new output variable to the replicated model to assess whether it can reproduce the pattern. In the case of reproduction, we argue that the original model is sufficient to produce the pattern, further strengthening its external validity [12]. If the replicated model does not reproduce the observed results, extra efforts are required to disentangle the reasons for this. Specifically, extensions incorporating new model components stemming from intuition, empirical observations, analytical models, or general theories are usually applied to implement a potential mechanism [19]. However, the objective remains to specify the mechanism likely responsible for the pattern. Once the pattern is reproduced, large-scale simulation experiments are used to identify the core driving variables and boundary conditions through a robustness analysis [20]. This ability supports a better understanding of the empirical pattern and provides insights into the likely mechanism behind the pattern [10, 12].

# References

1. Burman LE, Reed WR, Alm J. A call for replication studies. Public Finance Review. 2010;38(6):787-93.

2. Wilensky U, Rand W. Making models match: Replicating an agent-based model. Journal of Artificial Societies and Social Simulation. 2007;10(4):1-2.

3. Sansores C, Pavón J. Agent-based simulation replication: A model driven architecture approach. In: Gelbukh A, de Albornoz Á, Terashima-Marín H, editors. MICAI 2005: Advances in Artificial Intelligence; 2005. Berlin, Heidelberg: Springer Berlin Heidelberg; 2005. p. 244-53.

4. Robinson S. Conceptual modelling for simulation part i: Definition and requirements. Journal of the Operational Research Society. 2008;59(3):278-90.

5. Gary P, Luis RI, Nicholas MG. The ghost in the model (and other effects of floating point arithmetic). Journal of Artificial Societies and Social Simulation. 2004;8(1).

6. Anand V, Balakrishnan R, Labro E. A framework for conducting numerical experiments on cost system design. Journal of Management Accounting Research. 2019;31(1):41-61.

7. Edmonds B, Hales D. Replication, replication and replication: Some hard lessons from model alignment. Journal of Artificial Societies and Social Simulation. 2003;32(1).

8. Will O, Hegselmann R. A replication that failed on the computational model in 'michael w. Macy and yoshimichi sato: Trust, cooperation and market formation in the u.S. And japan. Proceedings of the national academy of sciences, may 2002'. Journal of Artificial Societies and Social Simulation. 2008;11(3):3.

9. Tivnan BF. Modeling organizational adaptation: A replication of levinthal’s model of emergent order. 2007 Winter Simulation Conference2007. p. 1241-6.

10. Thiele JC, Grimm V. Replicating and breaking models: Good for you and good for ecology. Oikos. 2015;124(6):691-6.

11. Fachada N, Lopes VV, Martins RC, Rosa AC. Model-independent comparison of simulation output. Simulation Modelling Practice and Theory. 2017;72:131-49.

12. Grimm V, Revilla E, Berger U, Jeltsch F, Mooij WM, Railsback SF, et al. Pattern-oriented modeling of agent-based complex systems: Lessons from ecology. Science. 2005;310(5750):987-91.

13. Heine B-O, Meyer M, Strangfeld O. Stylised facts and the contribution of simulation to the economic analysis of budgeting. Journal of Artificial Societies and Social Simulation. 2005;8(4):4.

14. Axtell R, Axelrod R, Epstein JM, Cohen MD. Aligning simulation models: A case study and results. Comput Math Organiz Theor. 1996;1(2):123-41.

15. Axelrod R. Advancing the art of simulation in the social sciences. In: Conte R, Hegselmann R, Terna P, editors. Complexity: Springer Berlin Heidelberg; 1997. p. 21-40.

16. Secchi D, Seri R. Controlling for false negatives in agent-based models: A review of power analysis in organizational research. Comput Math Organiz Theor. 2017;23(1):94-121.

17. White JW, Rassweiler A, Samhouri JF, Stier AC, White C. Ecologists should not use statistical significance tests to interpret simulation model results. Oikos. 2014;123(4):385-8.

18. Belding TC. Numerical replication of computer simulations: Some pitfalls and how to avoid them. Center for the Study of Complex Systems. 2000.

19. Balakrishnan R, Hansen S, Labro E. Evaluating heuristics used when designing product costing systems. Management Science. 2011;57(3):520-41.

20. Grimm V, Berger U. Robustness analysis: Deconstructing computational models for ecological theory and applications. Ecological Modelling. 2016;326:162-7.
