# Supplementary material for: Cost hierarchies and the pattern of product cost cross-subsidization: Extending a computational model of costing system design
Source: PLoS One. 2023 Sep 11;18(9):e0290370. doi: 10.1371/journal.pone.0290370 (PMC10495028; doi:10.1371/journal.pone.0290370)
Supplement: S2 Appendix — (DOCX) [file pone.0290370.s003.docx]

**S2 Appendix**

**Additional analysis: Kolmorogov-Smirnov-Test with increasing sample sizes**

The graph shows that with an increasing sample size the mean difference between regression coefficients of the original and replicated model decreases. Relational equivalence thus increases, because the effects of regression coefficients on the dependent variable (MAPE) become more similar, as stochastic matters less with more observations. Contrarily, the p-value for the Kolmogorov-Smirnov Test also decreases with larger sample-sizes. A smaller p-value indicates significant differences in distributional equivalence. This highlights that statistical significancy tests, such as the Kolmogorov-Smirnov-Test (KS-Test), are flawed for large sample sizes [1].


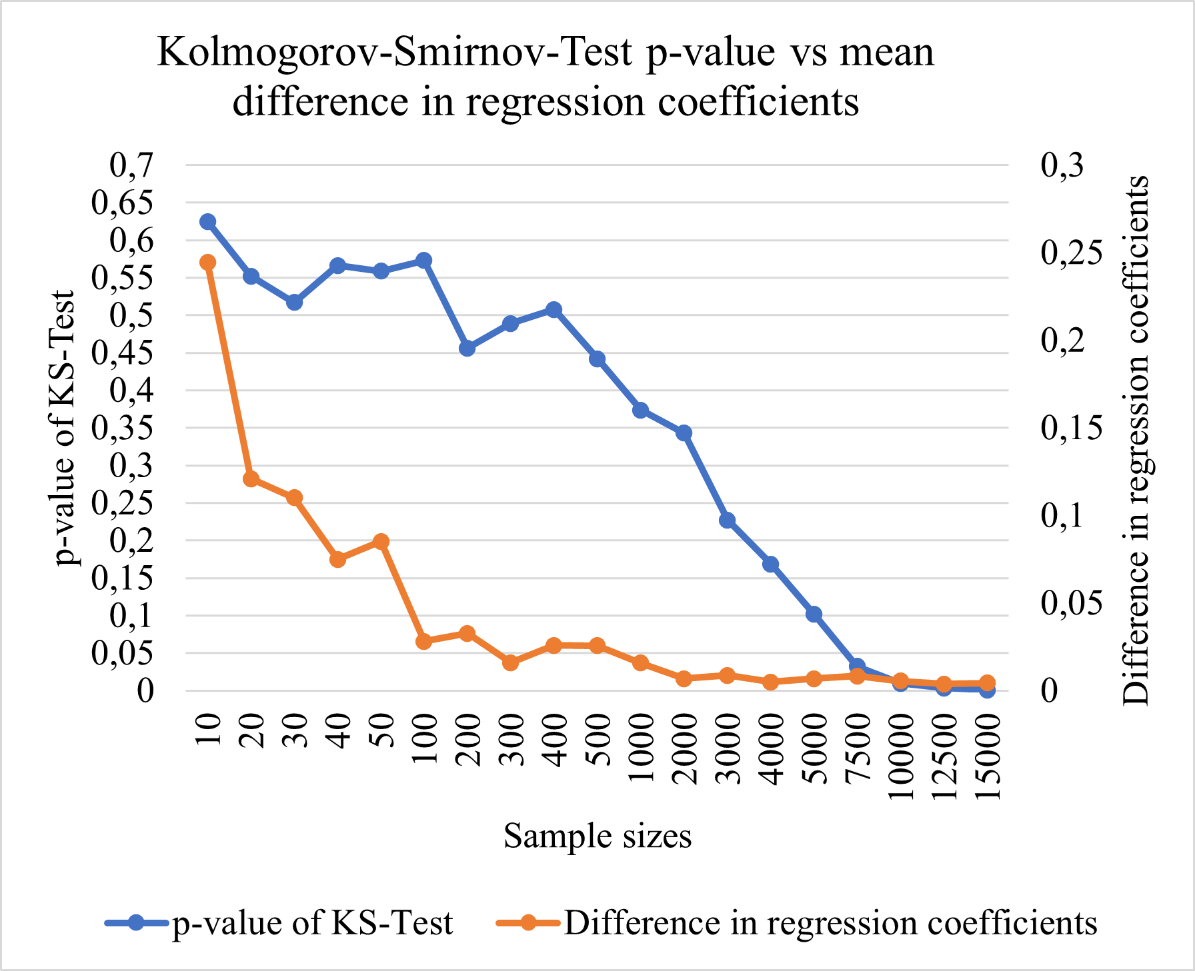

**KS-Test p-value vs. mean difference in regression coefficients.**

**References**

1. Secchi D, Seri R. Controlling for false negatives in agent-based models: a review of power analysis in organizational research. Comput Math Organiz Theor. 2017;23(1):94-121. doi: 10.1007/s10588-016-9218-0.
