# Supplementary material for: Cost hierarchies and the pattern of product cost cross-subsidization: Extending a computational model of costing system design
Source: PLoS One. 2023 Sep 11;18(9):e0290370. doi: 10.1371/journal.pone.0290370 (PMC10495028; doi:10.1371/journal.pone.0290370)
Supplement: S3 Appendix — (DOCX) [file pone.0290370.s004.docx]

**S3 Appendix**

**Additional analysis: Correlation between resource consumption and production volumes**

The graph shows that in the unchanged ABL framework the median correlation between resource consumption (i.e., columns in *RES_CONS_PATp*) and production volumes (*MXQ*) is above 0.75. This correlation decreases with more non-unit-level costs, thus highlighting that the multiplication of resource consumption with production volumes leads to unit-level resource consumption.


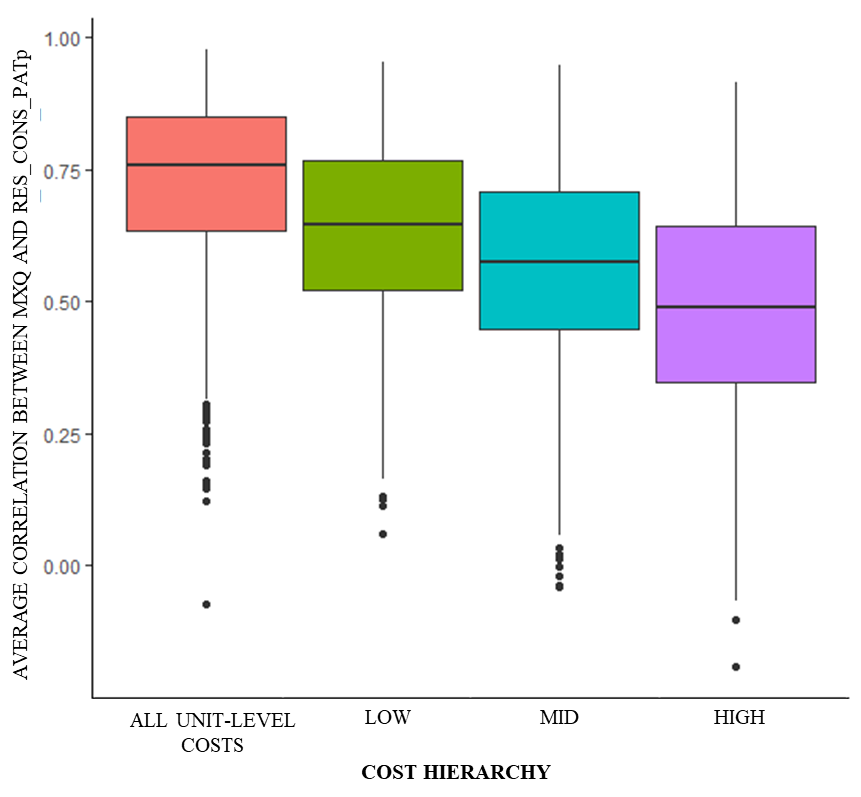


**Fig 11. Correlation between production volumes and resource consumption.** LOW = Share of costs that are consumed on the non-unit-level = 20% - 33%; MID = Share of costs that are consumed on the non-unit-level = 34% - 46%; HIGH = Share of costs that are consumed on the non-unit-level = 46%-60%.
