# Supplementary material for: Cost hierarchies and the pattern of product cost cross-subsidization: Extending a computational model of costing system design
Source: PLoS One. 2023 Sep 11;18(9):e0290370. doi: 10.1371/journal.pone.0290370 (PMC10495028; doi:10.1371/journal.pone.0290370)
Supplement: S4 Appendix — (DOCX) [file pone.0290370.s005.docx]

**S5 Appendix.** Overview of variables and technical terms in the simulation experiments.

| **Variable name/ Technical term** | **Description** | **Explanation** |
| --- | --- | --- |
| *ACT_CONS_PAT* | *Activity consumption matrix* | Contains information about the resource consumption as measured by the costing system for each cost driver. |
| *BE_AB* | *Difference between the share of significantly overcosted and the share of undercosted products* | Measures whether there are more products materially undercosted or overcosted (i.e., more than 5% difference to a product’s true costs). |
| *bl_size* | *Batch-level size* | The share of batch-level resources and costs, such as set-ups, maintenance, etc. |
| *CC* | *Correlation Cut-off variable* | The minimum similarity (correlation) in consumption between two resources to be allocated together in a cost pool. |
| *COR1* | *Correlation between volume resources* | Defines the correlation between resource consumption on the unit-level (in the original model). |
| *COR2* | *Correlation between batch resources* | Defines the correlation between resource consumptions on the batch-level (in the original model). |
| *CP* | *Number of Cost Pools* | Number of cost pools a firm employs to allocate indirect costs to individual cost objects. |
| *DENS* | *Density of the resource consumption matrix (RES_CONS_PAT)* | Defines the degree of resource sharing between all products in the product portfolio. Higher degrees of resource sharing may reflect a mass-production process where product variants differ only marginally [1]. Low degrees of resource sharing may reflect highly specialized single-unit production with high cost traceability [2]. |
| *DISP1* | *Number of "big” resources* | Defines the number of “big” resources. “Big” resources reflect high costs that carry a large fraction of total costs. For instance, this may be the total salary costs in labor-intensive production. |
| *DISP2* | *Share of costs that are assigned to the “big” resources* | Defines the share of total costs that fall onto the “big” resources and hence defines the centralization of the firm’s production processes on a few resources. |
| *fl_size* | *Facility-sustaining-level size* | The share of resources and costs that are facility-sustaining-level, such as CEO pay or facility rent. |
| *MISCPOOLSIZE* | *Relative share of costs in MISCPOOL* | Share of total costs that are allocated to the miscellaneous cost pool. The miscellaneous cost pool reflects a cost pool that is not directly associated with a specific group of activities or resources and contains resources that cannot be allocated to other cost pools and are thus grouped together [3]. |
| *MXQ* | *Production quantities per product* | Defines the production quantities (i.e., realized demand) of single products in the observed period. |
| *Non_unit_size* | *Non-unit-level size* | Share of resources that is *not* on the unit-level. In other words, resources that are not consumed in the production of single units. |
| *NUMB_PRO* | *Number of products* | Number of individual products in the product portfolio. |
| *NUMB_RES* | *Number of resources* | Number of individual resources (costs) consumed by the firm. |
| *PACP* | *Heuristic to allocate resources into cost pools* | Defines the heuristics chosen to group resources into cost pools. For instance, one approach may be to group similar resources into one cost pool (e.g., all marketing activities and costs into the “marketing pool”). The heuristics are described in the body of the paper or in the appendix of the ABL framework [3]. |
| *PCB* | *Benchmark costs of a cost object* | The true costs of individual products, based on the actual resource consumption of an individual product. Firms can only calculate product costs based on the simplified costing system (PCH) and therefore possess no information about these true costs (PCB). |
| *PCH* | *Heuristics costs of a cost object* | Reflects the product costs reported by the costing system and is therefore based on the simplified calculation. These costs deviate from the true costs (PCB) because a costing system only captures an aggregated and simplified picture of the true resource consumption [4]. |
| *PDR* | *Heuristic for cost-driver selection* | Defines the heuristic for cost driver selection for each cost pool. As described, a cost pool may contain several resources that are grouped together (e.g. cost pool “marketing” containing different marketing activities). The heuristic describes the general rule after which one of the activities is chosen to be the cost driver (allocation base) for all resources in that cost pool. Hence a firm may only measure the usage of that cost driver [1]. |
| *Percentage Error (PE)* | *PE = (PCH – PCB)/PCB* | Reflects the percentage deviation between true costs and reported costs of a product. Again, because true costs (PCB) are not attainable in empirical settings, the percentage error PE can only be calculated in artificial settings, such as a simulation experiment. |
| *pl_size* | *Product-sustaining-level size* | The share of resources and costs that are product-sustaining-level, such as product design activities, manufacturing planning, etc. |
| *Q_VAR* | *Diversity in production quantities* | Defines the disparity between production volumes of different products. Individual product variants may be produced in different quantities, with some products being so-called “high-runner” products and others being exotic, highly customized products [5]. The former may likely be produced in larger quantities than the latter. Q_VAR defines the disparity between such different product variants. |
| *RCC* | *Resource costs* | The resource cost vector contains the overall costs for all individual resources of the firm. This information may be available from financial accounting [6] in a firm’s financial statements. |
| *RES_CONS_PAT* | *Resource consumption pattern matrix* | The resource consumption matrix contains full and true information about the resource consumption of every single resource by every product. The resource consumption may reflect the actual and full activities and production process, or bill of materials [7] that arise to produce each product once, based on the production quantities MXQ [3]. |
| *TC* | *Total Costs* | The total costs of a firm that arise when producing the quantities given in MXQ. Can also be seen as the total costs from a firm’s financial statement. |
| *ul_size* | *Unit-level size* | The share of resources and costs that are unit-level, such as direct labor, machine hours, etc. |
| *VB_PATTERN* | *Variable for the product cost cross-subsidization pattern.* | The employed variable to measure the strength of the product cost cross-subsidization pattern. The larger the value for this variable, the greater the overcosting (undercosting) bias for high (low) volume products. |
| *VolumeDriver* | *Indicator variable or the employed type of cost driver* | Indicates which type of cost driver is employed in a costing system. The type of cost driver primarily defines whether a costing system is seen as an ABC system or as a volume-based costing system [8]. |

**References**

1. Balakrishnan R, Hansen S, Labro E. Evaluating heuristics used when designing product costing systems. Management Science. 2011;57(3):520-41.

2. Kerremans M, Theunisse H, Van Overloop G. Impact of automation on cost accounting. Accounting and Business Research. 1991;21(82):147-55.

3. Anand V, Balakrishnan R, Labro E. A framework for conducting numerical experiments on cost system design. Journal of Management Accounting Research. 2019;31(1):41-61.

4. Datar SM, Gupta M. Aggregation, specification and measurement errors in product costing. The Accounting Review. 1994;69(4):567.

5. Elmaraghy H, Schuh G, Elmaraghy W, Piller F, Schönsleben P, Tseng M, et al. Product variety management. CIRP Annals. 2013;62(2):629-52.

6. Balakrishnan R, Labro E, Sivaramakrishnan K. Product costs as decision aids: An analysis of alternative approaches (part 1). Accounting Horizons. 2012;26(1):1-20.

7. Anand V, Balakrishnan R, Gavirneni S. Capacity planning with limited information. Production and Operations Management. 2023;n/a(n/a).

8. Labro E. Analytics of costing system design. Contemporary issues in management accounting. 2006:217-42.
