# Supplementary material for: Cost hierarchies and the pattern of product cost cross-subsidization: Extending a computational model of costing system design
Source: PLoS One. 2023 Sep 11;18(9):e0290370. doi: 10.1371/journal.pone.0290370 (PMC10495028; doi:10.1371/journal.pone.0290370)
Supplement: S5 Appendix — (DOCX) [file pone.0290370.s006.docx]

**S6 Appendix.** Supporting information for data and code availability.

The source-code for the replicated simulation model and the generated datasets can be found here: https://doi.org/10.5281/zenodo.8248553

The Online Appendix contains the following files:

1. "*ABL_replication.zip*": The source code that was used to replicate the original model from Anand, Balakrishnan & Labro (2019).
2. "*ABL.csv*": The dataset generated with the ABL framework for the replication experiments.
3. "*REPLICATION.csv*": The dataset generated with the replicated simulation model for the replication experiments.
4. "*EXTENSION_1.csv*": The dataset generated with the replicated simulation model for the first extension numerical experiment containing a simple cost hierarchy and a volume-based cost driver.
5. "*EXTENSION_2.csv*": The dataset generated with the replicated simulation model for the second extension numerical experiment containing the theoretical and empirical ABC cost hierarchy.
